# ﻿Taxonomic novelties and global biogeography of *Montagnula* (Ascomycota, Didymosphaeriaceae)

**DOI:** 10.3897/mycokeys.101.113259

**Published:** 2024-01-19

**Authors:** Dhanushka N. Wanasinghe, Thilina S. Nimalrathna, Li Qin Xian, Turki Kh. Faraj, Jianchu Xu, Peter E. Mortimer

**Affiliations:** 1 Honghe Center for Mountain Futures, Kunming Institute of Botany, Chinese Academy of Sciences, Honghe County 654400, Yunnan, China Kunming Institute of Botany, Chinese Academy of Sciences Honghe County China; 2 CAS Key Laboratory of Tropical Forest Ecology, Xishuangbanna Tropical Botanical Garden, Chinese Academy of Sciences, Menglun, Mengla, Yunnan, China CAS Key Laboratory of Tropical Forest Ecology, Xishuangbanna Tropical Botanical Garden, Chinese Academy of Sciences Mengla China; 3 Southeast Asia Biodiversity Research Institute, Chinese Academy of Sciences & Center for Integrative Conservation, Xishuangbanna Tropical Botanical Garden, Chinese Academy of Sciences, Mengla, Yunnan 666303, China Southeast Asia Biodiversity Research Institute, Chinese Academy of Sciences & Center for Integrative Conservation, Xishuangbanna Tropical Botanical Garden, Chinese Academy of Sciences Mengla China; 4 Yunnan International Joint Laboratory of Southeast Asia Biodiversity Conservation & Yunnan Key Laboratory for Conservation of Tropical Rainforests and Asian Elephants, Menglun, Mengla, Yunnan 666303, China Yunnan International Joint Laboratory of Southeast Asia Biodiversity Conservation & Yunnan Key Laboratory for Conservation of Tropical Rainforests and Asian Elephants Mengla China; 5 International College, University of Chinese Academy of Sciences, Beijing, China University of Chinese Academy of Sciences Beijing China; 6 Department of Soil Science, College of Food and Agriculture Sciences, King Saud University, P.O. Box 145111, Riyadh 11362, Saudi Arabia King Saud University Riyadh Saudi Arabia; 7 CIFOR-ICRAF China Country Program, Kunming, Yunnan, China CIFOR-ICRAF China Country Program Kunming China

**Keywords:** Global distribution, microfungi, molecular phylogeny, taxonomy, Yunnan

## Abstract

Whilst conducting surveys of lignicolous microfungi in Yunnan Province, we collected a large number of taxa that resemble *Montagnula* (Didymosphaeriaceae, Pleosporales). Our phylogenetic study on *Montagnula* involved analysing sequence data from ribosomal RNA genes (nc18S, nc28S, ITS) and protein-coding genes (*rpb*2, *tef*1-α). We present a biphasic approach (morphological and molecular phylogenetic evidence) that supports the recognition of four new species in *Montagnula viz*., *M.lijiangensis*, *M.menglaensis*, *M.shangrilana* and *M.thevetiae*. The global diversity of *Montagnula* is also inferred from metabarcoding data and published records based on field observations. Metabarcoding data from GlobalFungi and field observations provided insights into the global diversity and distribution patterns of *Montagnula*. Studies conducted in Asia, Australia, Europe, and North America revealed a concentration of *Montagnula* species, suggesting regional variations in ecological preferences and distribution. *Montagnula* species were found on various substrates, with sediments yielding a high number of sequences. Poaceae emerged as a significant contributor, indicating a potential association between *Montagnula* species and grasses. Culture-based investigations from previously published data revealed *Montagnula* species associations with 105 plant genera (in 45 plant families), across 55 countries, highlighting their wide ecological range and adaptability. This study enhances our understanding of the taxonomy, distribution, and ecological preferences of *Montagnula* species. It emphasizes their role in the decomposition of organic matter in grasslands and savannah systems and suggests further investigation into their functional roles in ecosystem processes. The global distribution patterns and ecological interactions of *Montagnula* species underscore the need for continued research and conservation efforts.

## ﻿Introduction

Fungi are the second largest group of eukaryotes, performing vital ecological functions such as decomposition, mutualism, and pathogenesis to plants and animals (Tedersoo et al. 2014). Ascomycota, which forms the largest phylum of Fungi, and includes the genus *Montagnula*, is an incredibly diverse group, with an estimated global species richness of ~154,500 species (Bánki et al. 2023). Despite their ecological and economic importance, many Ascomycota species remain undescribed, and their distribution and diversity have yet to be properly determined (Maharachchikumbura et al. 2021a, b; Wijayawardene et al. 2022). This is somewhat due to the fact that many Ascomycota species are microscopic and inconspicuous, making them difficult to find and subsequently study, or sometimes these smaller species can be overlooked with studies focussing on more charismatic species of macrofungi (Wanasinghe et al. 2022a). The investigation of taxonomic and phylogenetic systematics in Ascomycota is bridging crucial knowledge gaps and enhancing our understanding of this particular group of fungi. *Montagnula* (typified with *M.infernalis*), is an example of a relatively understudied genus within Ascomycota, and many species remain undescribed. Understanding the taxonomic, phylogenetic and host relationships between *Montagnula* species will help us better understand how they have diversified and adapted to different habitats in various ecological zones. These data are useful to make predictions about the ecology and biology of the genus and to guide future research into their interactions with other organisms and their roles in ecosystem processes. Understanding the taxonomy and phylogeny of *Montagnula* is also important for conservation purposes. With ongoing habitat destruction and climate change, it is more important than ever to understand the current diversity and distribution of fungi around the world (Wanasinghe et al. 2022a).

Therefore, our research group at the Center for Mountain Futures (CMF), has been conducting investigations into the microfungal diversity and biogeography in Yunnan Province, Southwest China. Specifically, we are focusing on various substrates such as leaf and woody litter, aiming to clarify the taxonomy of fungi on these substrates, using morphology in conjunction with multigene phylogeny. As a result, we have successfully isolated numerous anamorphic and teleomorphic Ascomycota species in Yunnan, and we have published our findings based on different themes, including their relationship with hosts, substrates, and localities (Thiyagaraja et al. 2019, 2020, 2021; Abeywickrama et al. 2020; Wanasinghe et al. 2020, 2021, 2022b, 2023; Yasanthika et al. 2020; Bundhun et al. 2021; Dissanayake et al. 2021; Gao et al. 2021; Monkai et al. 2021; Mortimer et al. 2021; Ren et al. 2021a, b, 2022a, b; Aluthmuhandiram et al. 2022; Maharachchikumbura et al. 2022; Wanasinghe and Mortimer 2022). The objectives of this study are (1) to identify the lignicolous *Montagnula* species collected from Yunnan using both morphological and phylogenetic approaches, and (2) to utilize metabarcoding data and published records based on field observations to infer the global diversity and biogeography of *Montagnula*. The analyses conducted in this study revealed four new species and four existing species of *Montagnula*, in Yunnan. The discovery of several previously undescribed Ascomycota species in the genus *Montagnula* in Yunnan Province is a significant advancement in our understanding of the diversity and distribution of this group of fungi. Furthermore, the utilization of metabarcoding data and published records based on field observations to infer the global diversity of *Montagnula* demonstrates the potential of these approaches in elucidating the biogeography of fungi on a large scale. By studying and documenting the diversity of *Montagnula* species, we can enhance our appreciation for the importance of conserving these fungi and their habitats, and take appropriate measures to mitigate the threats they face.

## ﻿Materials and methods

### ﻿Sample collecting

Fresh fungal materials were collected from dead woody twigs from Honghe, Kunming, Mengla, Shangri-La and Yulong Counties, all within Yunnan Province, China, during the dry season (January, March, April) and wet season (August, September). To preserve their integrity, the specimens were transported to the laboratory in Zip lock plastic bags during the dry season and in paper bags during the wet season.

### ﻿Morphological observations

The morphology of external and internal macro-/micro-structures were observed as described in Wanasinghe et al. (2017, 2018a, 2020). Hand sections of the ascomata were mounted in distilled water and the following characteristics were evaluated and measured: ascomata diameter, height, color and shape; width of peridium; and height and diameter of ostioles. Length and width (at the widest point) of asci and ascospores. Images were captured with a Canon EOS 600D digital camera fitted to a Nikon ECLIPSE Ni compound microscope. Macroscopic images of colonies were documented using an iPhone XS Max (Apple Inc., Cupertino, CA, USA) with daylight. Measurements were made with the Tarosoft (R) Image Frame Work program, and images used for figures were processed with Adobe Photoshop CS5 Extended version 10.0 software (Adobe Systems, San José, CA, USA).

### ﻿Isolation

Single spore isolation was conducted by following the methods described in Wanasinghe et al. (2018b). Germinated spores were individually transferred to potato dextrose agar (PDA: 39 g/L distilled water, Difco potato dextrose) plates and grown at 20 °C in the daylight.

### ﻿Deposition of specimens, cultures and registering names

The living cultures were deposited at the Kunming Institute of Botany Culture Collection (KUNCC), Kunming, China. Dry herbarium materials were deposited in the herbarium of Cryptogams Kunming Institute of Botany, Academia Sinica (KUN-HKAS). MycoBank numbers have been obtained as outlined in MycoBank (http://www.MycoBank.org accessed on 21 September 2023) for the novel taxa.

### ﻿DNA extraction, PCR amplifications and sequencing

Genomic DNA was extracted from the axenic mycelium as described by Phookamsak et al. (2017). Mycelia for DNA extraction from each isolate were grown on PDA for 3–4 weeks at 20 °C and total genomic DNA was extracted from approximately 150 ± 50 mg axenic mycelium scraped from the edges of the growing culture. Mycelium was ground to a fine powder with liquid nitrogen and DNA extracted using the Biospin Fungus Genomic DNA Extraction Kit-BSC14S1 (BioFlux, P.R. China) following the instructions of the manufacturer. When fungi failed to grow in culture, DNA extraction was carried out directly from fruiting bodies, adhering to the protocol outlined by Wanasinghe et al. (2018b). DNA to be used as templates for Polymerase Chain Reaction (PCR) were stored at 4 °C for use in regular work and duplicated at -20 °C for long-term storage.

We used primers ITS5/ITS4 (White et al. 1990), LR0R/LR5 (Vilgalys and Hester 1990; Rehner and Samuels 1994), NS1/NS4 (White et al. 1990), EF1-983F/EF1-2218R (Liu et al. 1999; Rehner and Buckley 2005), and fRPB2-5f/fRPB2-7cR (Sung et al. 2007) to amplify sequence data for a total of five markers: the internal transcribed spacers (ITS), partial 28S large subunit rDNA (LSU), partial 18S small subunit rDNA (SSU), translation elongation factor 1-α (*tef*1-α), and RNA polymerase II second largest subunit (*rpb*2). PCR amplifications were performed following the methods described in Wanasinghe et al. (2021). We sequenced complementary strands with the same primers used for PCR amplifications and sequencing was done from a commercial sequencing provider (BGI, Ltd Shenzhen, P.R. China). The nucleotide sequence data obtained were deposited in GenBank (Table 2).

### ﻿Sequencing assembly and alignments

Sequences generated from different primers of the five genes were analysed with other sequences retrieved from GenBank (Table 2). Sequences with high similarity indices were determined from a BLAST search to find the closest matches with taxa in Didymosphaeriaceae, using recently published data (Du et al. 2021; Ren et al. 2022a; Sun et al. 2023). The multiple alignments of all consensus sequences, as well as the reference sequences were automatically generated with MAFFT v. 7 (Katoh et al. 2019), and manually corrected where necessary using BioEdit v. 7.0.5.2 (Hall 1999).

### ﻿Phylogenetic inference

The single-locus datasets were examined for topological incongruence among loci for members of the analyses. The alignments were concatenated into a multi-locus alignment that was analyzed with maximum likelihood (ML) and Bayesian (BI) phylogenetic methods in the CIPRES Science Gateway (Miller et al. 2010). ML tree was obtained using RAxML-HPC2 on XSEDE v. 8.2.10 (Stamatakis 2014) with applying GTR+G+I model. Support values were obtained with 1,000 bp replicates (Felsenstein 1985). ML bootstrap values equal or greater than 75% are given above each node. The best-fit model was selected with respect to Bayesian Information Criterion (BIC) scores using the IQ-TREE web application at http://iqtree.cibiv.univie.ac.at (Trifinopoulos et al. 2016). For model selection, we restricted the pool of available models to JC, F81, HKY, SYM and GTR (Ronquist et al. 2011). BI were performed with two parallel runs of 2 M generations, using four chains in each, and retaining one tree every 100 generations. The dataset was partitioned by gene region, and a GTR + G + I model was applied to each partition, ending the run automatically when standard deviation of split frequencies dropped below 0.01 with a burn-in fraction of 0.25. A fifty percent majority rule consensus tree was obtained after discarding the first 25% of trees, and posterior probabilities were used as a measure of nodal support. The posterior probability in BI (BYPP) greater than 0.95 are given above each node. Phylograms were visualized with FigTree v1.4.0 program (Rambaut 2012) and reorganized in Microsoft power point (2019).

### ﻿The biogeographical distribution of *Montagnula*

In our initial approach, we obtained detailed geographical distribution information for the *Montagnula* genus. This data was extracted from the GlobalFungi database (https://globalfungi.com, accessed on 04 December 2023), as outlined by Větrovský et al. (2020). The database provided information on the countries and precise geographical coordinates of recorded *Montagnula* occurrences. To visualize these occurrences, we employed a range of packages in R version 4.2.1 (R Core Team 2022), including ‘sf’ (Pebesma and Bivand 2023), ‘raster’ (Hijmans 2023), ‘rgdal’ (Bivand et al. 2022), and ‘ggplot2’ (Wickham 2011). In our map, each marker signifies an individual occurrence of *Montagnula*. These occurrences are visually distinguished by a color scheme, with each color denoting the specific biome from which the samples were collected, as illustrated in Fig. 2a. Additionally, we have developed two donut charts, showcased in Fig. 2b, c, which effectively illustrate the distribution of *Montagnula* sequences. These charts present the sequence abundance as a percentage of the total, segmented across various biomes and continents, providing a clear visual breakdown of their distribution. Furthermore, we have gathered Environmental DNA (eDNA) data from diverse sources in metabarcoding studies focusing on fungi, as found in the GlobalFungi database (Fig. 3). This dataset included specifics about eDNA sources, locations of the studies, and the sequence abundance of *Montagnula* sequences. It is important to note that the sequence abundance in metabarcoding studies might not always accurately represent the actual abundance of species in a habitat. Nonetheless, these data can provide valuable insights into the potential rarity or prevalence of the group in the eDNA source. We analyzed the sequence abundance in diverse eDNA samples from different continents. Before visualization, the abundance values were normalized via a logarithmic transformation to ensure a standardized and comparable presentation of *Montagnula* sequence abundance. Post-transformation abundance data were visualized using the ‘ggplot2’ package, aiding in highlighting the focus areas of metabarcoding and identifying the environmental sample types from which *Montagnula* sequences were derived across various continents (Figs 2, 3).

### ﻿The host relations of *Montagnula*

To illustrate the host specificity of *Montagnula* species, we utilized detailed information regarding host species from the literature (Table 1). This enabled us to create informative bar plots displaying the host preferences of *Montagnula* species (Fig. 4). This information was visualized using the ‘ggplot2’ package in R.

**Table 1.** Accepted species in *Montagnula* including their host and geographic location.

Species	Host species	Host family	Country	Reference
*Montagnulaacaciae*	*Acaciaauriculiformis*	Fabaceae	Thailand	Tennakoon et al. (2022) ^#^
*Montagnulaaloes*	*Aloe* sp.	Asphodelaceae	South Africa	Crous et al. (2012) ^#^
*Montagnulaappendiculata*	*Zeamays*	Poaceae	China	Aptroot (2004) ^#^
*Montagnulaaquatica*	Submerged wood	NA	Thailand	Sun et al. (2023) ^#^
	Dead woody litter	NA	China	This study^#^
*Montagnulaaquilariae*	*Aquilariasinensis*	Thymelaeaceae	China	Hyde et al. (2023) ^#^
	Dead woody litter	NA	China	This study^#^
*Montagnulabaatanensis*	*Agave* sp.	Asparagaceae	USA	Crivelli (1983)
*Montagnulabellevaliae*	*Bellevaliaromana*	Asparagaceae	Italy	Hongsanan et al. (2015) ^#^
*Montagnulacamporesii*	*Dipsacus* sp.	Caprifoliaceae	Italy	Hyde et al. (2020) ^#^
*Montagnulacamarae*	*Cytisusscoparius*	Fabaceae	Portugal	Checa (2004)
*Montagnulachiangraiensis*	*Chromolaenaodorata*	Asteraceae	Thailand	Mapook et al. (2020) ^#^
*Montagnulachromolaenae*	*Chromolaenaodorata*	Asteraceae	Thailand	Mapook et al. (2020) ^#^
*Montagnulachromolaenicola*	*Chromolaenaodorata*	Asteraceae	Thailand	Mapook et al. (2020) ^#^
	*Lagerstroemia* sp.	Lythraceae	China	This study^#^
*Montagnulacirsii*	*Cirsium* sp.	Asteraceae	Italy	Hyde et al. (2016) ^#^
*Montagnulacylindrospora*	Human skin^##^	NA	USA	Crous et al. (2020) ^#^
*Montagnuladasylirionis*	*Dasylirion* sp.	Asparagaceae	USA	Ramaley and Barr (1995)
*Montagnuladonacina*	*Acaciareficiens*	Fabaceae	Namibia	Aptroot (1995)
	*Acacia* sp.	Fabaceae	India	Aptroot (1995)
	*Adhatodavasica*	Acanthaceae	India	Aptroot (1995)
	*Ailanthusaltissima*	Simaroubaceae	India	Aptroot (1995)
	*Althaearosea*	Malvaceae	China	Aptroot (1995)
	*Annonasquamosa*	Annonaceae	India	Aptroot (1995)
	*Arundodonax*	Poaceae	Portugal	Aptroot (1995)
	*Bambusoideae*	Poaceae	Brazil	Aptroot (1995)
	*Bambusoideae*	Poaceae	Papua New Guinea	Aptroot (1995)
	*Cajanuscajan*	Fabaceae	India	Aptroot (1995)
	*Calamusaustralis*	Arecaceae	Australia	Hyde et al. (1999)
	*Careyaarborea*	Lecythidaceae	India	Aptroot (1995)
	*Citrusaurantiifolia*	Rutaceae	India	Aptroot (1995)
	*Clerodendruminfortunatum*	Lamiaceae	India	Aptroot (1995)
	*Clerodendrummultiflorum*	Lamiaceae	India	Aptroot (1995)
	*Coffeaarabica*	Rubiaceae	Paraguay	Aptroot (1995)
	*Coffearobusta*	Rubiaceae	Central African Republic	Aptroot (1995)
	*Craterellusodoratus* ^##^	Cantharellaceae	China	Zhao et al. (2018) ^#^
	*Durantarepens*	Verbenaceae	India	Aptroot (1995)
	*Ficusglomerata*	Moraceae	India	Aptroot (1995)
	*Funtumiaafricana*	Apocynaceae	Sierra Leone	Aptroot (1995)
	*Hibiscus* sp.	Malvaceae	India	Aptroot (1995)
	*Ipomoeacarnea*	Convolvulaceae	India	Aptroot (1995)
	*Mallotusphilippinensis*	Euphorbiaceae	India	Aptroot (1995)
	*Morusalba*	Moraceae	India	Aptroot (1995)
	*Litchilitchi*	Sapindaceae	Myanmar	Thaung (2008)
*Montagnuladonacina*	*Neriumodorum*	Apocynaceae	India	Aptroot (1995)
	*Paeoniasuffruticosa*	Paeoniaceae	China	Li et al. (2023) ^#^
	*Phyllostachysbambusoides*	Poaceae	Japan	Wang et al. (2004)
	*Pistacia* sp.	Anacardiaceae	India	Aptroot (1995)
	*Platanus* sp.	Platanaceae	USA	Wang et al. (2004)
	*Premnacumingiana*	Lamiaceae	Philippines	Aptroot (1995)
	*Pseudosasajaponica*	Poaceae	France	Aptroot (1995)
	*Saccharumofficinarum*	Poaceae	Brazil	Aptroot (1995)
	Unknown stem	NA	India	Aptroot (1995)
	*Tectonagrandis*	Lamiaceae	India	Aptroot (1995)
	*Terminaliatomentosa*	Combretaceae	India	Aptroot (1995)
	*Trachycarpusfortunei*	Arecaceae	China	Hyde et al. (1999)
	Unknown bark	NA	India	Aptroot (1995)
	Unknown branches	NA	Sierra Leone	Aptroot (1995)
	Unknown plant	NA	Colombia	Aptroot (1995)
	Dead wood	NA	China	Sun et al. (2023) ^#^
	Dead wood	NA	Thailand	Ren et al. (2022a) ^#^
	Dead wood	NA	China	This study^#^
	*Vitisvinifera*	Vitaceae	Australia	Pitt et al. (2014) ^#^
	*Wikstroemia* sp.	Thymelaeaceae	USA	Aptroot (1995)
	*Zeamays*	Poaceae	Georgia	Aptroot (1995)
*Montagnuladura*	*Aconitumseptentrionale*	Ranunculaceae	Sweden	Eriksson (1992)
	*Loniceraetrusca*	Caprifoliaceae	Spain	Checa (2004)
*Montagnulagilletiana*	*Fraxinusornus*	Oleaceae	Bulgaria	Fakirova (2004)
	*Retamasphaerocarpa*	Fabaceae	Spain	Checa (2004)
	*Ulexeuropaeus*	Fabaceae	Spain	Checa (2004)
*Montagnulagraminicola*	Poaceae	Poaceae	Italy	Liu et al. (2015) ^#^
*Montagnulaguiyangensis*	*Helwingiahimalaica*	Helwingiaceae	China	Sun et al. (2023) ^#^
*Montagnulahirtula*	*Cerastiumlatifolium*	Caryophyllaceae	Austria	Leuchtmann (1984)
	*Cerastium* sp.	Caryophyllaceae	Italy	Leuchtmann (1984)
	*Epilobiumparviflorum*	Onagraceae	Switzerland	Leuchtmann (1984)
	*Rubusidaeus*	Rosaceae	Finland	Leuchtmann (1984)
	*Rubus* sp.	Rosaceae	Sweden	Eriksson (1992)
*Montagnulainfernalis*	*Agaveamericana*	Asparagaceae	Portugal	Checa (2004)
	*Agaveamericana*	Asparagaceae	Spain	Checa (2004)
	*Fourcroya* sp.	Asparagaceae	Portugal	Ariyawansa et al. (2014)
	*Furcraeagigantea*	Asparagaceae	Portugal	Checa (2004)
	*Furcraeagigantea*	Asparagaceae	Spain	Checa (2004)
	*Furcraealongaeva*	Asparagaceae	Portugal	Checa (2004)
	*Furcraealongaeva*	Asparagaceae	Spain	Checa (2004)
*Montagnulainfernalis*	*Furcraeamacrophylla*	Asparagaceae	Bahamas	Barr (1990)
*Montagnulajonesii*	*Fagussylvatica*	Fagaceae	Italy	Tennakoon et al. (2016) ^#^
	*Ficusbenjamina*	Moraceae	Thailand	Tennakoon et al. (2022) ^#^
*Montagnulakrabiensis*	*Pandanus* sp.	Pandanaceae	Thailand	Tibpromma et al. (2018) ^#^
*Montagnulalijiangensis*	*Quercus* sp.	Fagaceae	China	This study^#^
*Montagnulalongipes*	*Agaveamericana*	Asparagaceae	Algeria	Aptroot (1995)
*Montagnulamelanorhabdos*	*Agave* sp.	Asparagaceae	Turkey	Aptroot (2006)
*Montagnulamenglaensis*	*Indocalamustessellatus*	Poaceae	China	This study^#^
*Montagnulamohavensis*	*Yuccamohavensis*	Asparagaceae	USA	Ramaley and Barr (1995)
*Montagnulaobtusa*	*Ilex* sp.	Aquifoliaceae	USA	French (1989)
	*Juglans* sp.	Juglandaceae	USA	French (1989)
	*Pinuspinaster*	Pinaceae	Portugal	Checa (2004)
	*Sorbusaucuparia*	Rosaceae	Sweden	Eriksson (1992)
*Montagnulaopaca*	*Phalaris*	Poaceae	Switzerland	Crivelli (1983)
*Montagnulaopulenta*	*Ammophilaarenaria*	Poaceae	France	Aptroot (1995)
	*Ammophilaarenaria*	Poaceae	Germany	Aptroot (1995)
	*Ammophilaarenaria*	Poaceae	Sweden	Aptroot (1995)
	*Festucabrachyphylla*	Poaceae	Canada	Aptroot (1995)
	*Opuntiaficus-indica*	Cactaceae	Canary Islands	Aptroot (1995)
	*Opuntiaficus-indica*	Cactaceae	France	Aptroot (1995)
	*Opuntiaficus-indica*	Cactaceae	Italy	Aptroot (1995)
	*Opuntiaficus-indica*	Cactaceae	Malta	Aptroot (1995)
	*Opuntiaficus-indica*	Cactaceae	Tunisia	Aptroot (1995)
	*Opuntia* sp.	Cactaceae	Cyprus	Aptroot (1995)
	*Opuntia* sp.	Cactaceae	Israel	Aptroot (1995)
	*Opuntia* sp.	Cactaceae	Italy	Aptroot (1995)
	*Opuntia* sp.	Cactaceae	Tunisia	Aptroot (1995)
	*Opuntiatuna*	Cactaceae	USA	Aptroot (1995)
	*Poaabbreviata*	Poaceae	Canada	Aptroot (1995)
	*Puccinelliaangustata*	Poaceae	Greenland	Aptroot (1995)
	*Stipahimalaica*	Poaceae	India	Aptroot (1995)
*Montagnulaopuntiae*	*Opuntialindheimeri*	Cactaceae	USA	Huhndorf (1992)
*Montagnulapalmacea*	*Chamaeropshumilis*	Arecaceae	France	Aptroot (1995)
	*Cocoscapitata*	Arecaceae	Spain	Aptroot (1995)
	*Daviesianudiflora*	Fabaceae	Australia	Aptroot (1995)
	*Phoenixdactylifera*	Arecaceae	Egypt	Aptroot (1995)
	*Phoenixdactylifera*	Arecaceae	Greece	Aptroot (1995)
	*Phoenixdactylifera*	Arecaceae	Iraq	Aptroot (1995)
	*Phoenixdactylifera*	Arecaceae	Italy	Aptroot (1995)
	*Phoenixdactylifera*	Arecaceae	Pakistan	Aptroot (1995)
	*Phoenixdactylifera*	Arecaceae	Saudi Arabia	Aptroot (1995)
	*Phoenixdactylifera*	Arecaceae	Tunisia	Aptroot (1995)
	*Phoenixsylvestris*	Arecaceae	Pakistan	Aptroot (1995)
	*Pitcairniachrysantha*	Bromeliaceae	Chile	Aptroot (1995)
	Unknown leaves	NA	USA	Aptroot (1995)
	Unknown petiole	NA	USA	Aptroot (1995)
*Montagnulaperforans*	*Calamagrostisarenaria*	Poaceae	France	Aptroot (2006)
*Montagnulaphragmospora*	*Agaveamericana*	Asparagaceae	Portugal	Checa (2004)
	*Agaveamericana*	Asparagaceae	Spain	Checa (2004)
	*Agavehookeri*	Asparagaceae	Portugal	Checa (2004)
	*Agavehookeri*	Asparagaceae	Spain	Checa (2004)
	*Agave* sp.	Asparagaceae	France	Barr (1990)
	*Agave* sp.	Asparagaceae	Portugal	Checa (2004)
	*Agave* sp.	Asparagaceae	Spain	Checa (2004)
*Montagnulaphragmospora*	*Yuccabrevifolia*	Asparagaceae	California	Barr (1990)
	*Yucca* sp.	Asparagaceae	Portugal	Checa (2004)
	*Yucca* sp.	Asparagaceae	Spain	Checa (2004)
*Montagnulapuerensis*	Dead wood	NA	China	Du et al. (2021) ^#^
*Montagnularhodophaea*	*Arundodonax*	Poaceae	Italy	Leuchtmann (1984)
	*Phragmitescommunis*	Poaceae	Switzerland	Leuchtmann (1984)
*Montagnulasaikhuensis*	*Citrus* sp.	Rutaceae	Thailand	Wanasinghe et al. (2016) ^#^
*Montagnulascabiosae*	*Scabiosa* sp.	Caprifoliaceae	Italy	Hongsanan et al. (2015) ^#^
*Montagnulashangrilana*	*Rhododendron* sp.	Ericaceae	China	This study^#^
*Montagnula* sp.	*Carexfuliginosa*	Cyperaceae	Austria	Scheuer (1988)
*Montagnulaspartii*	*Aeluropuslittoralis*	Poaceae	Russia	Aptroot (1995)
	*Ammophilaarenaria*	Poaceae	Belgium	Aptroot (1995)
	*Ammophilaarenaria*	Poaceae	Denmark	Aptroot (1995)
	*Ammophilaarenaria*	Poaceae	Sweden	Aptroot (1995)
	*Ammophilaarenaria*	Poaceae	United Kingdom	Aptroot (1995)
	*Calamagrostisepigeios*	Poaceae	Russia	Aptroot (1995)
	*Calycotomespinosa*	Fabaceae	France	Aptroot (1995)
	*Calycotomespinosa*	Fabaceae	Spain	Aptroot (1995)
	*Calycotomevillosa*	Fabaceae	Italy	Aptroot (1995)
	*Carexrostrata*	Cyperaceae	Sweden	Aptroot (1995)
	*Chamaeropshumilis*	Arecaceae	Spain	Aptroot (1995)
	*Leymusarenarius*	Poaceae	Russia	Aptroot (1995)
	*Ephedraciliata*	Ephedraceae	Unknown country in Asia	Aptroot (1995)
	*Ephedra* sp.	Ephedraceae	Iran	Aptroot (1995)
	*Festucaarenaria*	Poaceae	France	Aptroot (1995)
	*Festucasulcata*	Poaceae	Iran	Aptroot (1995)
	*Genistaaspalathoides*	Fabaceae	Italy	Aptroot (1995)
	*Gramineae*	Gramineae	Austria	Aptroot (1995)
	*Koeleriacristata*	Poaceae	Germany	Aptroot (1995)
	*Koeleriaglauca*	Poaceae	Denmark	Aptroot (1995)
	*Linumaustriacum*	Linaceae	Germany	Aptroot (1995)
	*Luzulaspadicea*	Juncaceae	Switzerland	Aptroot (1995)
	*Lygeumspartum*	Poaceae	Spain	Aptroot (1995)
	*Melicaciliata*	Poaceae	France	Aptroot (1995)
	*Nardusstricta*	Poaceae	Austria	Aptroot (1995)
	*Puccinelliapeisonis*	Poaceae	Austria	Aptroot (1995)
	*Sarothamnusscoparius*	Fabaceae	Poland	Mulenko et al. (2008)
	*Sarothamnusscoparius*	Fabaceae	Switzerland	Aptroot (1995)
	*Sesleriacaerulea*	Poaceae	Italy	Aptroot (1995)
*Montagnulaspartii*	*Spartiumjunceum*	Fabaceae	Albania	Aptroot (1995)
	*Spartiumjunceum*	Fabaceae	France	Aptroot (1995)
	*Spartiumjunceum*	Fabaceae	Greece	Aptroot (1995)
	*Spartiumjunceum*	Fabaceae	Turkey	Aptroot (1995)
	*Ulex* sp.	Fabaceae	Spain	Aptroot (1995)
*Montagnulaspinosella*	*Abeliatriflora*	Caprifoliaceae	Spain	Checa (2004)
	*Carexaterrima*	Cyperaceae	Austria	Scheuer (1988)
*Montagnulaspinosella*	*Carexmisandra*	Cyperaceae	Norway	Holm and Holm (1993, 1994)
	*Colpodiumvahlianum*	Poaceae	Norway	Holm and Holm (1993, 1994)
	*Deschampsiacaespitosa*	Poaceae	Norway	Holm and Holm (1993, 1994)
	*Juncusmaritimus*	Juncaceae	Spain	Holm and Holm (1993), Checa (2004)
	*Luzulaconfusa*	Juncaceae	Norway	Holm and Holm (1993, 1994)
*Montagnulastromatosa*	*Phoenixhanceana*	Arecaceae	China	Lu et al. (2000)
	*Phoenix* sp.	Arecaceae	China	Zhuang (2001)
	*Trachycarpusfortunei*	Arecaceae	China	Hyde et al. (1999)
	*Trachycarpusfortunei*	Arecaceae	United Kingdom	Hyde et al. (1999)
*Montagnulasubsuperficialis*	*Panicumgrumosum*	Poaceae	Argentina	Shoemaker (1989)
*Montagnulathailandica*	*Chromolaenaodorata*	Asteraceae	Thailand	Mapook et al. (2020) ^#^
	*Heveabrasiliensis*	Euphorbiaceae	Thailand	Senwanna et al. (2021) ^#^
	Coffeaarabicavar.catimor	Rubiaceae	China	Lu et al. (2022) ^#^
	Unidentified twig	NA	Thailand	Boonmee et al. (2021) ^#^
*Montagnulathevetiae*	*Thevetiaperuviana*	Apocynaceae	China	This study^#^
*Montagnulathuemeniana*	*Yucca* sp.	Asparagaceae	USA	Barr (1990)
*Montagnulatriseti*	*Trisetumdistichophyllum*	Poaceae	Switzerland	Crivelli (1983)
*Montagnulavakrabeejae*	Unidentified twig	NA	Andaman	Niranjan and Sarma (2018)
*Montagnulaverniciae*	*Verniciafordii*	Euphorbiaceae	China	Li et al. (2023) ^#^
*Montagnulayuccigena*	*Yuccabaccata*	Asparagaceae	Mexico	Ramaley and Barr (1995)

“^#^” Denotes molecular data available in GenBank. “^##^” Denotes none plant based. NA represents not applicable.

**Table 2.** GenBank accession numbers of sequences used for the phylogenetic analyses.

Taxon	Strain number	GenBank accession numbers					Reference
		ITS	LSU	SSU	*tef*1-α	*rpb*2	
*Montagnulaacaciae*	MFLUCC 18-1636	ON117280	ON117298	ON117267	ON158093	NA	Tennakoon et al. (2022)
	NCYUCC 19-0087^T^	ON117281	ON117299	ON117268	ON158094	NA	Tennakoon et al. (2022)
*Montagnulaaloes*	CPC 19671	JX069863	JX069847	NA	NA	NA	Crous et al. (2012)
	CBS 132531^T^	NR_111757	NG_042676	NA	NA	NA	Crous et al. (2012)
*Montagnulaappendiculata*	CBS 109027^T^	DQ435529	AY772016	NA	NA	NA	Wanasinghe et al. (2016)
*Montagnulaaquatica*	MFLU 22-0171^T^	OP605992	OP605986	OP600504	NA	NA	Sun et al. (2023)
***Montagnulaaquatica***	**KUNCC 23-14425**	**OR583097**	**OR583116**	**OR583135**	**OR588088**	**OR588107**	**This study**
	**KUNCC 23-14557**	**OR583099**	**OR583118**	**OR583137**	**OR588090**	**OR588109**	**This study**
*Montagnulaaquilariae*	KUNCC 22-10815^T^	OP452927	OP482265	OP482268	OP426318	NA	Hyde et al. (2023)
	KUNCC 22-10816	OP554219	OP482266	OP482269	OP426319	NA	Hyde et al. (2023)
	KUNCC 22-10815^T^	OP452927	OP482265	OP482268	OP426318	NA	Hyde et al. (2023)
	KUNCC 22-10816	OP554219	OP482266	OP482269	OP426319	NA	Hyde et al. (2023)
***Montagnulaaquilariae***	**KUNCC 23-14430**	**OR583100**	**OR583119**	**OR583138**	**OR588091**	**OR588110**	**This study**
	**KUNCC 23-14431**	**OR583101**	**OR583120**	**OR583139**	**OR588092**	**OR588111**	**This study**
	**KUNCC 23-14432**	**OR583102**	**OR583121**	**OR583140**	**OR588093**	**OR588112**	**This study**
*Montagnulabellevaliae*	MFLUCC 14-0924^T^	KT443906	KT443902	KT443904	NA	NA	Hongsanan et al. (2015)
*Montagnulacamporesii*	MFLUCC 16-1369^T^	MN401746	NG_070946	NG_068418	MN397908	MN397909	Hyde et al. (2020)
*Montagnulachiangraiensis*	MFLUCC 17-1420^T^	NR_168864	NG_068707	NG_070155	NA	NA	Mapook et al. (2020)
*Montagnulachromolaenae*	MFLUCC 17-1435^T^	NR_168865	NG_068708	NG_070156	NA	NA	Mapook et al. (2020)
*Montagnulachromolaenicola*	MFLUCC 17-1469^T^	NR_168866	NG_070948	NG_070157	MT235773	MT235809	Mapook et al. (2020)
***Montagnulachromolaenicola***	**KUNCC 23-14426**	**OR583098**	**OR583117**	**OR583136**	**OR588089**	**OR588108**	**This study**
	**KUNCC 23-14427**	**OR583103**	**OR583122**	**OR583141**	**OR588094**	**OR588113**	**This study**
	**KUNCC 23-14558**	**OR583104**	**OR583123**	**OR583142**	**OR588095**	**OR588114**	**This study**
*Montagnulacirsii*	MFLUCC 13-0680	KX274242	KX274249	KX274255	KX284707	NA	Hyde et al. (2016)
*Montagnulacylindrospora*	CBS 146572^T^	LT796834	LN907351	NA	LT797074	LT796994	Crous et al. (2020)
*Montagnuladonacina*	HFG07004	MF967419	MF183940	NA	NA	NA	Zhao et al. (2017)
	HVVV01	KJ628375	KJ628377	KJ628376	NA	NA	Pitt et al. (2014)
	HKAS 124552	OP605991	OP605987	NA	NA	NA	Sun et al. (2023)
	KUMCC 21-0653	OP058961	OP059052	OP059003	OP135938	NA	Ren et al. (2021)
	KUMCC 21-0579	OP058963	OP059054	OP059005	OP135940	NA	Ren et al. (2021)
	KUMCC 21-0631	OP058962	OP059053	OP059004	OP135939	NA	Ren et al. (2021)
	UESTCC 23.0030	OR253120	OR253279	OR253194	NA	NA	Unpublished
***Montagnuladonacina***	**KUNCC 23-14428**	**OR583105**	**OR583124**	**OR583143**	**OR588096**	**OR588115**	**This study**
	**KUNCC 23-14429**	**OR583106**	**OR583125**	**OR583144**	**OR588097**	**OR588116**	**This study**
*Montagnulagraminicola*	MFLUCC 13-0352^T^	KM658314	KM658315	KM658316	NA	NA	Liu et al. (2015)
*Montagnulaguiyangensis*	HKAS 124556^T^	OP605989	OP600484	OP600500	NA	NA	Sun et al. (2023)
	GUCC 22–0817	OP605990	OP600485	OP600501	NA	NA	Sun et al. (2023)
*Montagnulajonesii*	MFLUCC 16-1448^T^	KY313619	KY273276	KY313618	KY313620	NA	Tennakoon et al. (2016)
	MFLU 18-0084	ON117282	ON117300	ON117269	ON158095	NA	Tennakoon et al. (2022)
*Montagnulakrabiensis*	MFLUCC 16-0250^T^	NR168179	NG068826	NG068385	MH412776	NA	Tibpromma et al. (2018)
***Montagnulalijiangensis***	**HKAS 126540**	**OR583107**	**OR583126**	**OR583145**	**OR588098**	**OR588117**	**This study**
	**HKAS 126541^T^**	**OR583108**	**OR583127**	**OR583146**	**OR588099**	**OR588118**	**This study**
***Montagnulamenglaensis***	**KUNCC 23-14422**	**OR583109**	**OR583128**	**OR583147**	**OR588100**	**OR588119**	**This study**
	**KUNCC 23-14423**	**OR583110**	**OR583129**	**OR583148**	**OR588101**	**OR588120**	**This study**
	**KUNCC 23-14424^T^**	**OR583111**	**OR583130**	**OR583149**	**OR588102**	**OR588121**	**This study**
*Montagnulapuerensis*	KUMCC 20-0225^T^	MW567739	MW575866	MW575864	MW575859	NA	Du et al. (2021)
	KUMCC 20-0331	MW567740	MW575867	MW575865	MW575860	NA	Du et al. (2021)
*Montagnulasaikhuensis*	MFLUCC 16-0315^T^	KU743209	KU743210	KU743211	NA	NA	Wanasinghe et al. (2016)
*Montagnulascabiosae*	MFLUCC 14-0954^T^	KT443907	KT443903	KT443905	NA	NA	Hongsanan et al. (2015)
***Montagnulashangrilana***	**KUNCC 23-14433**	**OR583112**	**OR583131**	**OR583150**	**OR588103**	**OR588122**	**This study**
	**KUNCC 23-14434^T^**	**OR583113**	**OR583132**	**OR583151**	**OR588104**	**OR588123**	**This study**
*Montagnulathailandica*	MFLUCC 17-0363	OL782142	OL782059	OL780525	OL875102	OL828754	Senwanna et al. (2021)
	MFLUCC 17-1508^T^	MT214352	NG070949	NG070158	MT235774	MT235810	Mapook et al. (2020)
	MFLUCC 21-0075	OP297807	OP297777	OP297791	OP321576	NA	Lu et al. (2022)
	ZHKUCC 22-0206	OP297808	OP297778	OP297792	OP321577	NA	Lu et al. (2022)
	ZHKUCC 22-0207	MZ538515	MZ538549	NA	MZ567092	NA	Boonmee et al. (2021)
***Montagnulathevetiae***	**HKAS 126963**	**OR583114**	**OR583133**	**OR583152**	**OR588105**	**OR588124**	**This study**
	**HKAS 126964^T^**	**OR583115**	**OR583134**	**OR583153**	**OR588106**	**OR588125**	**This study**
*Neokalmusiajonahhulmei*	KUMCC 21-0818^T^	ON007043	ON007039	ON007048	ON009133	ON009137	Wanasinghe and Mortimer (2022)
*Neokalmusiajonahhulmei*	KUMCC 21-0819	ON007044	ON007040	ON007049	ON009134	ON009138	Wanasinghe and Mortimer (2022)

Ex-type strains are indicated with superscript “T”, and newly generated sequence is shown in bold. NA represents sequences that are unavailable in GenBank. CBS: Culture Collection of the Westerdijk Fungal Biodiversity Institute, Netherlands; CPC: Personal collection of P.W. Crous, Netherlands; HFG: Personal collection of Zhen-Zhu Zhao; GUCC: Guizhou University Culture Collection (GUCC), Guiyang, China; HKAS/KUNCC: Kunming Institute of Botany Culture Collection, China; HVVV: Personal collection of Wayne Pitt from *Vitisvinifera*; MFLUCC/MFLU: Mae Fah Luang University Culture Collection, Chiang Rai, Thailand; NCYUCC: National Chiayi University Culture Collection, Taiwan, China; UESTCC: University of Electronic Science and Technology Culture Collection; ZHKUCC: Zhongkai University of Agriculture and Engineering Culture Collection.

## ﻿Results

### ﻿Phylogenetic analyses

**Figure 1.**
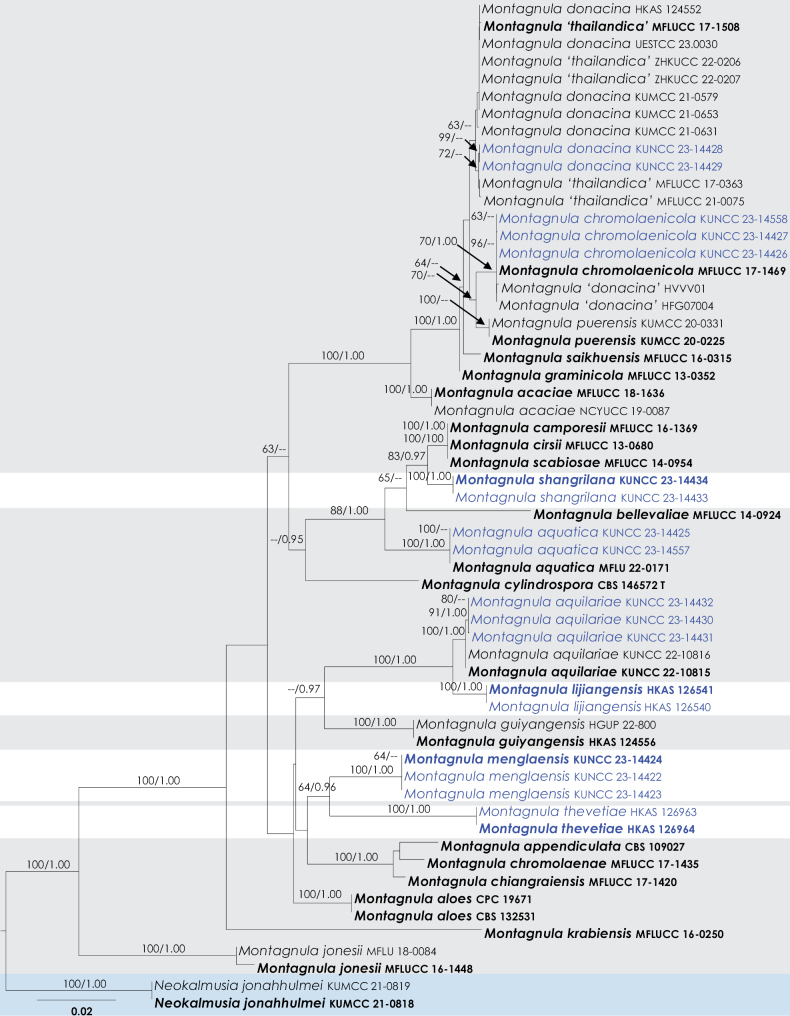
Phylogenetic analysis of SSU, LSU, ITS, *tef*1-α, and *rpb*2 of the *Montagnula*. Species names given in bold are ex-type, ex-epitype and ex-paratype strains. Species names highlighted in blue are generated from this study. Branch support of nodes ≥75% ML BS and ≥0.95 PP is indicated above the branches. The genus *Montagnula* is depicted within a pale gray box, with new species highlighted in white, and the outgroup indicated by a blue box.

In order to examine the evolutionary relationships of our new strains within *Montagnula*, phylogenetic analyses were performed based on the combined SSU, LSU, ITS, *tef*1-α, and *rpb*2 DNA sequences of 56 representatives of the genus and two strains from *Neokalmusiajonahhulmei* (KUMCC 21-0818, KUMCC 21-0819) as the outgroup taxon. The full dataset consisted of 4,268 characters including gaps (18S = 1,023 characters, 28S = 896, ITS = 508, *tef*1-α = 885, *rpb*2 = 956). The RAxML analysis of the combined dataset yielded a best-scoring tree with a final ML optimization likelihood value of -14,343.052271. The matrix had 1004 distinct alignment patterns, with 23.88% undetermined characters or gaps. Parameters for the GTR + I + G model of the combined amplicons were as follows: Estimated base frequencies; A = 0.244145, C = 0.256118, G = 0.269851, T = 0.229886; substitution rates AC = 1.815063, AG = 3.954334, AT = 1.414215, CG = 1.362941, CT = 10.779403, GT = 1.000; proportion of invariable sites I = 0.559204; and gamma distribution shape parameter α = 0.542439. The Bayesian analysis ran 1,675,000 generations before the average standard deviation for split frequencies reached below 0.01 (0.009994). The analyses generated 16,751 trees, from which we sampled 12,564 trees after discarding the first 25% as burn-in. The alignment contained a total of 1,005 unique site patterns. The BI and ML trees were not in conflict; the ML tree is shown in Fig. 1. Where applicable, the phylogenetic results obtained (Fig. 1) are discussed in the descriptive notes below.

**Figure 2.**
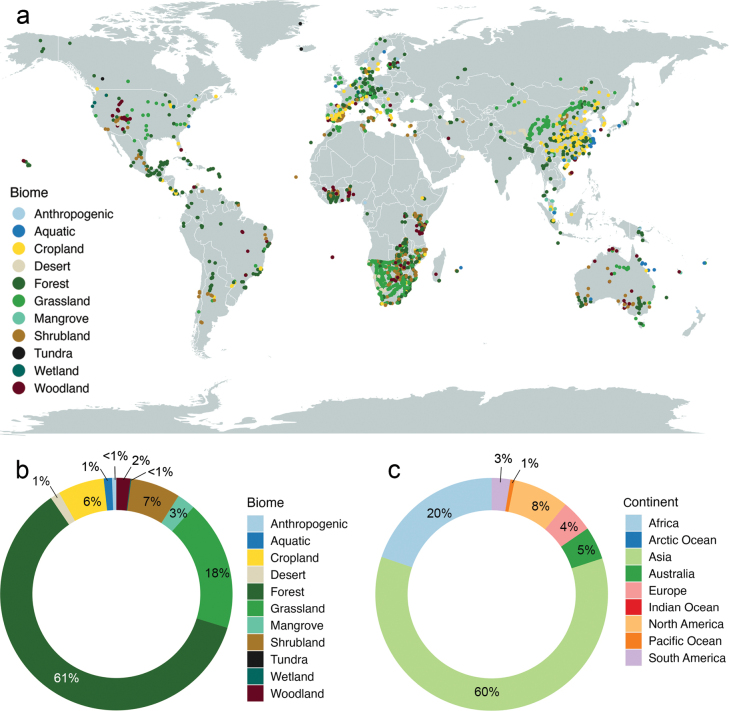
Geographical distribution of *Montagnula* species with known ITS sequence data. **a** the map summarizes data from the GlobalFungi database (shown by circles). Each circle symbolizes a unique sample, with each color representing the specific biome from which it has been collected **b** the distribution of *Montagnula* sequences as a percentage of total abundance across different biomes **c** the distribution of *Montagnula* sequences as a percentage of total abundance across different continents. See Suppl. material 1 for primary data.

**Figure 3.**
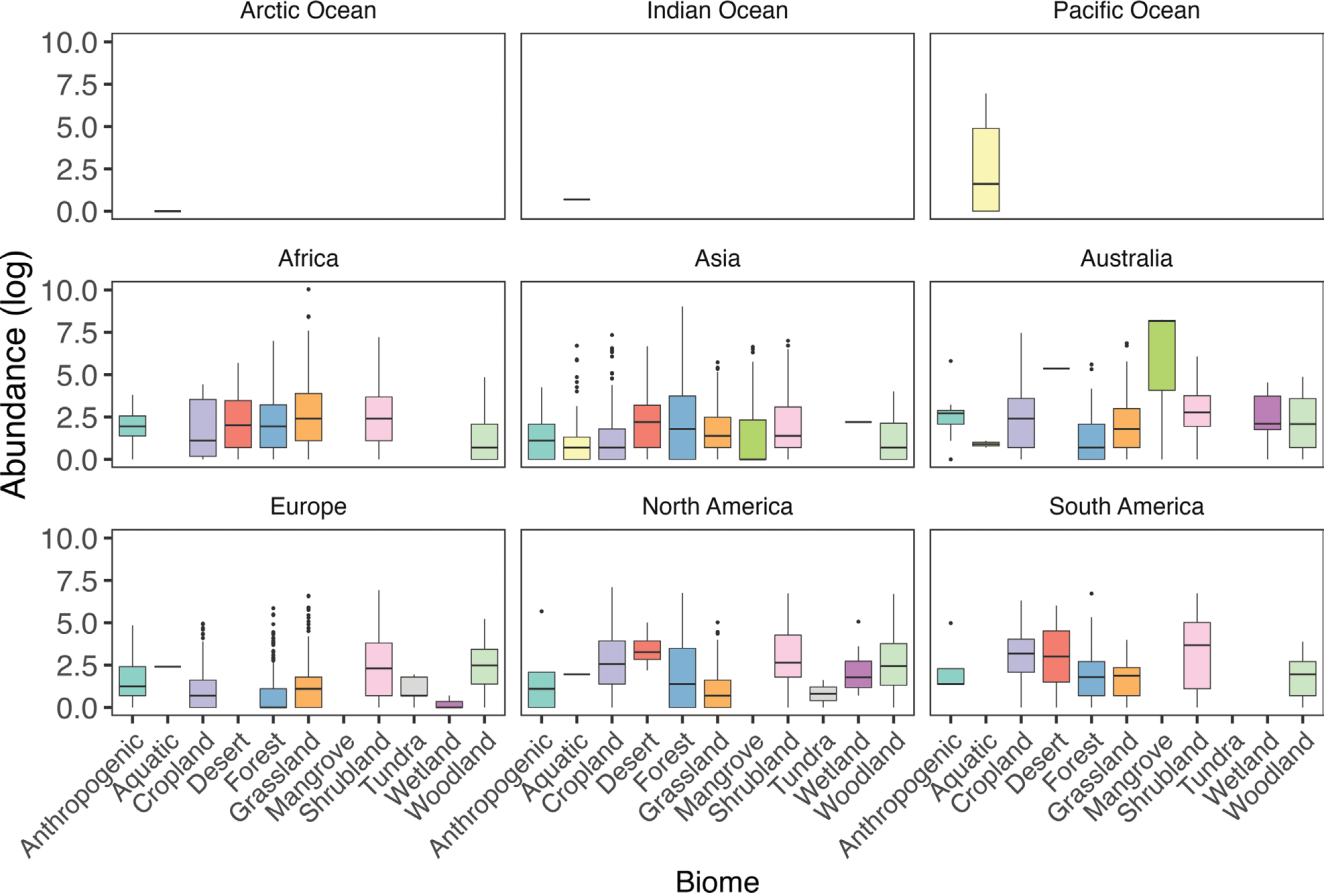
The distribution of *Montagnula* occurrences across oceans, continents and various substrates, as documented in the existing literature. On the x-axis, the logarithmic abundance of each record for different sources is displayed.

**Figure 4.**
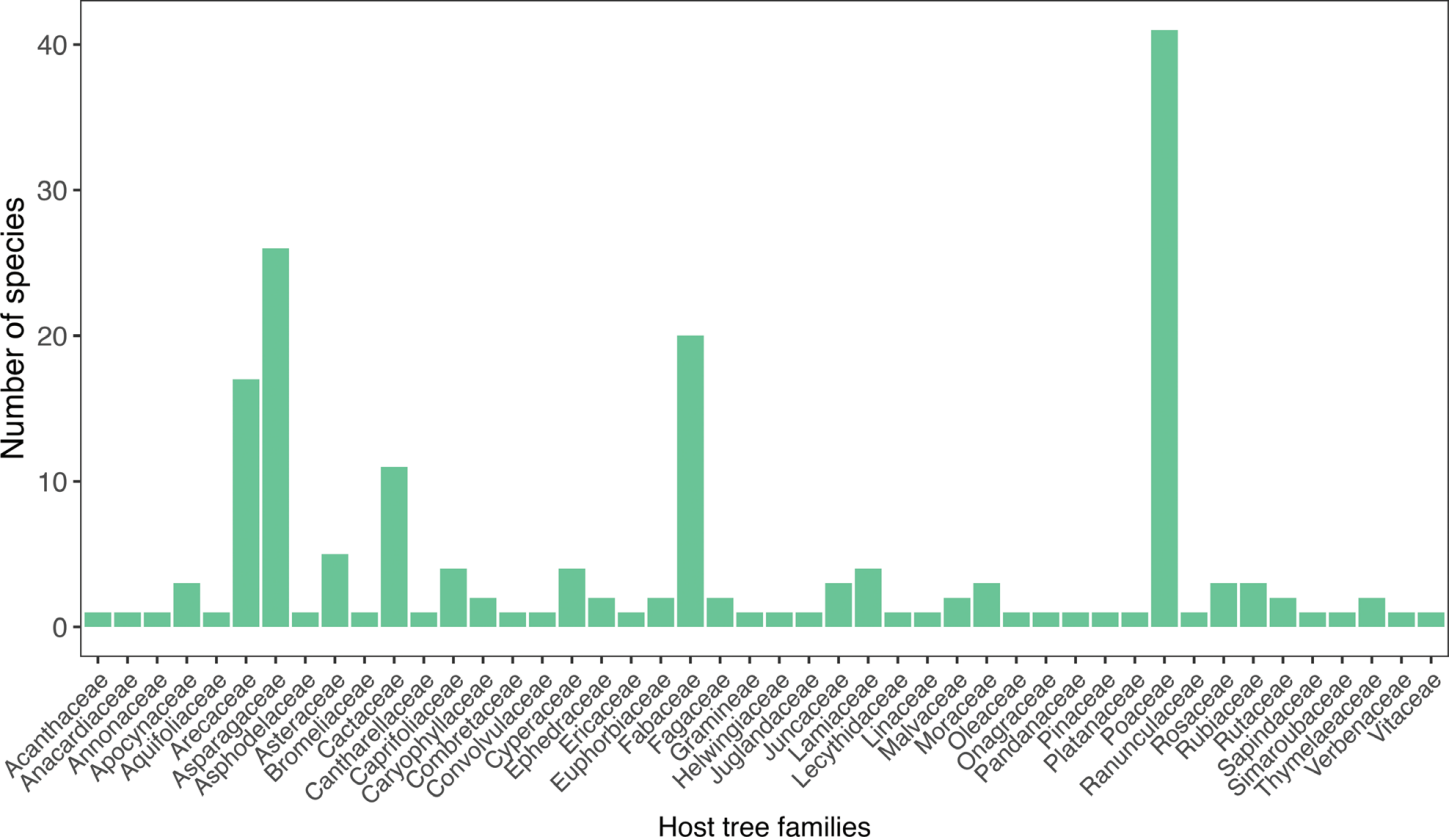
The species richness of recorded *Montagnula* species across different plant families (Table 1).

We conducted a thorough study of a compilation of data derived from multiple metabarcoding studies, which documented the occurrence of *Montagnula* species worldwide, excluding Antarctica. Among the continents, the highest number of studies were recorded in Asia, Australia, Europe, and North America (Fig. 2). These studies encompassed a diverse range of 11 distinct sources, revealing that sediments and “other” sources yielded the highest number of sequences (Fig. 3). Across different continents, the sequences obtained from various sources exhibited moderate similarity. However, in regions such as Asia, Australia, Europe, and North America, studies revealed *Montagnula* species from a diverse array of sources, in contrast to other studies, which identified species from a more limited selection of sources. Furthermore, in culture-based investigations, the primary focus was on extracting *Montagnula* species from plant substrates originating from 45 distinct plant families (Fig. 4). Among these families, Poaceae yielded the most substantial number of isolated species, followed by Asparagaceae and Fabaceae. Additionally, two records were also detected in mushrooms and human skin samples.

### ﻿Taxonomy

**Pleosporales Luttr. ex M.E. Barr, Prodromus to class Loculoascomycetes: 67 (1987)**

**Didymosphaeriaceae Munk, Dansk botanisk Arkiv 15 (2): 128 (1953)**

#### ﻿ Montagnula

Taxon classificationFungiPleosporalesDidymosphaeriaceae

Berl., Icones Fungorum. Pyrenomycetes 2: 68 (1896)

578C3145-D42A-50B7-B15C-424F97A756E7

##### Notes.

This study presents an updated and comprehensive phylogenetic classification of the genus *Montagnula*, incorporating SSU, LSU, ITS, *tef*1-α, and *rpb*2 DNA sequence analyses. By combining morphological and phylogenetic considerations, we have identified four new species, *M.lijiangensis*, *M.menglaensis*, *M.shangrilana* and *M.thevetiae* within the genus. Additionally, this research accounts for the existing species *viz.*, *M.aquatica*, *M.aquilariae*, *M.chromolaenicola* and *M.donacina*. The note sections of this publication provide detailed information on these taxonomic accounts, including additional discussion and supporting evidence. Each newly identified species adds to the known biodiversity within the genus, expanding our knowledge of the ecological and morphological characteristics exhibited by *Montagnula* taxa.

#### ﻿ Montagnula aquatica

Taxon classificationFungiPleosporalesDidymosphaeriaceae

Y.R. Sun, Yong Wang bis & K.D. Hyde, Plants 12 (4, no. 738): 2 (2023)

2F7D3DA1-0510-540B-9F7C-CD668E1655EF

900129

##### Descriptions and illustrations.

See Sun et al. (2023).

##### Habitat and distribution.

This species is found in freshwater habitats of Chiang Rai, Thailand, terrestrial habitats of Yunnan, China, inhabiting dead wood of deciduous hosts (Sun et al. 2023, this study).

##### Material examined.

China, Yunnan Province, Honghe Hani and Yi Autonomous Prefecture, Honghe County, Dayangjiexiang (23.389965°N, 102.225552°E, 1194 m), on dead woody litter of an unidentified plant, 13 March 2023, D.N. Wanasinghe, DWHH23-51 (HKAS 130322), new country and habitat record, living culture KUNCC 23-14425. *ibid*. 23.388966°N, 102.224786°E, 1215 m, DWHH23-51-2 (HKAS 130323), living culture KUNCC 23-14557.

##### Notes.

Based on our phylogenetic analyses, we have determined that the newly collected strains (i.e. KUNCC 23-14425 and KUNCC 23-14557) are monophyletic with the ex-type strain of *Montagnulaaquatica* (MFLU 22-0171). Further morphological investigations comparing our isolate with the type species have revealed similarities in the size range of the ascomata, asci, and ascospores, as well as the ascospore septation (Sun et al. 2023). Therefore, we document KUNCC 23-14425 and KUNCC 23-14557 as new records of *Montagnulaaquatica* in China, accompanied by protein sequence data (*tef*1-α and *rpb*2) for this species. It is worth noting that the holotype of *Montagnulaaquatica* was previously reported on submerged decaying wood in a freshwater habitat in Thailand, while our collection was made from a terrestrial habitat in China. This observation suggests that this fungus exhibits adaptability to a wide range of habitats, although its exploration in diverse geographic locations remains limited. The inclusion of *Montagnulaaquatica* as a new record in China expands our understanding of the distribution and ecological preferences of this species in both terrestrial and aquatic habitats. Additionally, the protein sequence data obtained for this strain contributes valuable information to the existing knowledge on *Montagnulaaquatica*. Further studies exploring the ecological aspects of this fungus in different geographic locations will provide deeper insights into its adaptability and potential ecological roles.

#### ﻿ Montagnula aquilariae

Taxon classificationFungiPleosporalesDidymosphaeriaceae

T.Y. Du & Tibpromma, Mycosphere 14 (1): 705 (2023)

15EC9B91-3FA0-566C-99B6-842140ED4B7B

846332

Fig. 5

##### Description.

***Saprobic*** on dead woody litter of an unknown deciduous host. **Teleomorph *Ascomata*** 450–600 μm high × 480–550 μm diam., immersed to semi-erumpent, gregarious or rarely clustered, globose to subglobose, ostiolate. ***Ostiole*** 120–220 × 70–110 µm (x– = 139 × 89 μm, n = 5), papillate, central, straight, dark brown to black, filled with hyaline cells, periphyses are lacking. ***Peridium*** 20–40 μm thick on the sides and can reach up to 60 μm near the apex, with an outer layer consisting of heavily pigmented cells that have thick walls and exhibit a ***textura angularis*** to ***textura globulosa*** texture at the apex, ***textura angularis*** texture at the sides and base; the innermost layer consists of narrow, hyaline compressed rows of cells that merge with pseudoparaphyses. ***Hamathecium*** of 2–4 μm broad, dense, narrow, branched, cellular pseudoparaphyses. ***Asci*** 100–120 × 16–22 µm (x– = 110.8 × 18.4 μm, n = 20), bitunicate, fissitunicate, cylindrical-clavate to clavate, pedicel 30–50 μm long, 8-spored, biseriate, with a minute ocular chamber best seen in immature ascus. ***Ascospores*** 20–25 × 8.5–11 µm (x– = 21.8 × 9.6 μm, n = 30), ellipsoidal to narrowly oblong, straight or somewhat curved, ends conically rounded, golden-brown to dark brown, 1-septate, constricted at the septum, large guttules in each cell, verruculose, with a thin mucilaginous sheath. **Anamorph** Undetermined.

##### Habitat and distribution.

This species is found in terrestrial habitats of Yunnan, China, specifically inhabiting dead woody twigs of deciduous hosts, including *Aquilariasinensis* (Hyde et al. 2023, this study).

##### Material examined.

China, Yunnan Province, Kunming City, Kunming Institute of Botany (25.141723°N, 102.750013°E, 1970 m), on dead woody litter of an unidentified plant, 24 April 2022, L. Qinxian, KIB22-17-1 (HKAS 126542), living culture KUNCC 23-14430; *ibid*. 25.141487°N, 102.748863°E, 1982 m, K2B22-17-3 (HKAS 126543), living culture KUNCC 23-14431; *ibid*. K2B22-17-4 (HKAS 126544), living culture KUNCC 23-14432.

##### Notes.

*Montagnulaaquilariae* was recently introduced by Hyde et al. (2023) based on samples obtained from *Aquilariasinensis* in Xishuangbanna, Yunnan Province. In our new collections, three strains (KUNCC 23-14430, KUNCC 23-14431, KUNCC 23-14432) exhibited a monophyletic relationship with the previously known strains of *Montagnulaaquilariae* (KUNCC 22-10815 [ex-type] and KUNCC 22-10816). Through further morphological, ecological, and nucleotide (SSU, LSU, ITS, *tef*1-α) comparisons, we have confirmed that these new strains indeed belong to *Montagnulaaquilariae*. Our research also provides additional insights into the characteristics of *Montagnulaaquilariae*. Specifically, we report the verruculose feature of the ascospores and present *rpb*2 sequence data for this fungus, advancing our knowledge of its morphological and genetic attributes.

**Figure 5.**
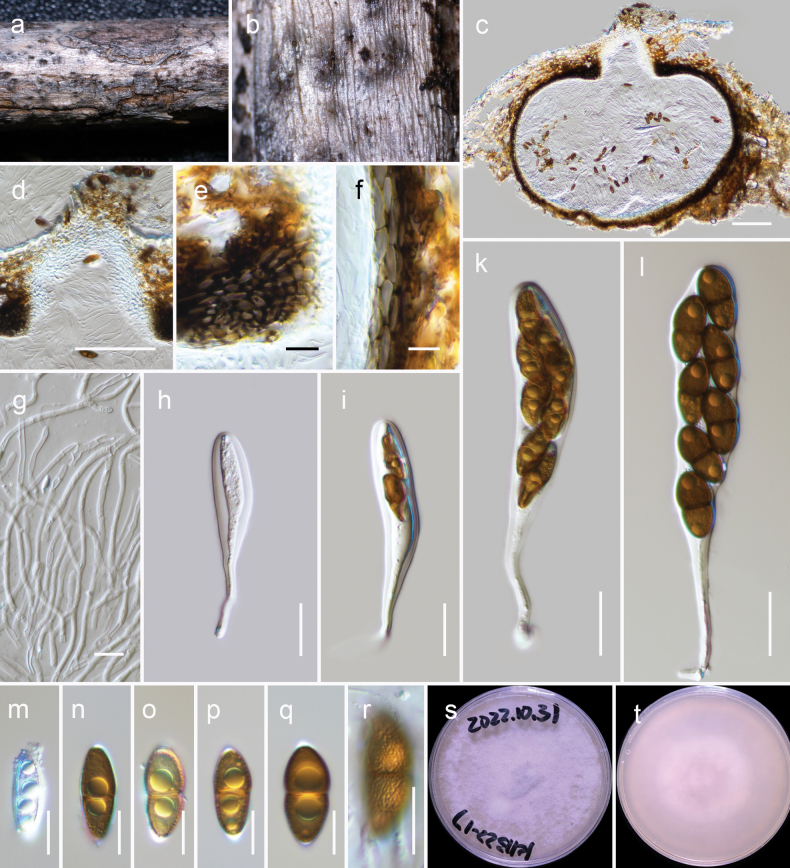
*Montagnulaaquilariae* (HKAS 126542) **a, b** ascomata on natural wood surface **c** vertical section through an ascoma **d** ostiolar neck **e** peridium cells at the apex **f** peridium cells at the side **g** pseudoparaphyses **h–l** asci **m–r** ascospores (see verruculose feature of the ascospore in r) **s, t** culture characters on PDA (s = above, t = reverse). Scale bars: 100 μm (**c, d**); 50 μm (**e**); 10 μm (**e–g, m–r**); 20 μm (**h–l**).

#### ﻿ Montagnula chromolaenicola

Taxon classificationFungiPleosporalesDidymosphaeriaceae

Mapook & K.D. Hyde, Fungal Diversity 101: 35 (2020)

F82A329C-CBA2-5A45-BBBD-B1CE8FD17D0A

557298

##### Descriptions and illustrations.

See Mapook et al. (2020).

##### Habitat and distribution.

This species was observed in terrestrial habitats in Mae Hong Son, Thailand, specifically on dead stems of *Chromolaenaodorata* (Mapook et al. 2020). Additionally, it has also been found in terrestrial habitats in Yunnan, China, where it inhabits dead wood of deciduous hosts (this study).

##### Material examined.

China, Yunnan Province, Honghe County, Honghe Hani and Yi Autonomous Prefecture, Dayangjiexiang (23.389965°N, 102.225552°E, 1201 m), on a dead woody climber of an unidentified host, 13 March 2023, D.N. Wanasinghe, DWHH23-17A (HKAS 130321), living culture KUNCC 23-14426. *ibid*. 23.389295°N, 102.224780°E, 1200 m, on dead twigs of *Lagerstroemia* sp. DWHH23-33-2 (HKAS 126543), living culture KUNCC 23-14427; *ibid*. DWHH23-33-3 (HKAS 130320), living culture KUNCC 23-14558.

##### Notes.

Through our phylogenetic analyses, we have determined that the newly isolated strains HH33 and HH17A exhibit a monophyletic relationship with the ex-type strain of *Montagnulachromolaenicola* (MFLUCC 17-1469). Upon conducting further investigations and morphological comparison of our collection with the type species, we have discovered several similarities. These include the size range of the ascomata, asci, and ascospores, as well as the ascospore septation (Mapook et al. 2020). Consequently, we hereby document our new collections (i.e. HKAS 130321, HKAS 126543 and HKAS 130320) as new records of *Montagnulachromolaenicola* in China. In a recent study by Sun et al. (2023), *Montagnulachromolaenicola*, *M.puerensis*, *M.saikhuensis*, and *M.thailandica* were synonymized under the name *M.donacina* due to the absence of obvious branches in their phylogenetic tree and the close morphological resemblance between these species. However, it is important to note that most of these strains lack informative sequence data for *tef*1-α or *rpb*2. Our observations, on the other hand, have revealed that the inclusion of protein data in this group leads to the formation of distinct branches and independent lineages. Therefore, we propose retaining the older names for these species, except for *Montagnulathailandica*, until further research resolves this group using all available sequence data.

#### ﻿ Montagnula donacina

Taxon classificationFungiPleosporalesDidymosphaeriaceae

(Niessl) Wanas., E.B.G. Jones & K.D. Hyde, Index Fungorum 319: 1 (2017)

1CC08730-61AE-54B9-A85C-5F8459AB30CC

552762

##### Descriptions and illustrations.

See Pitt et al. (2014).

##### Habitat and distribution.

This species has been reported worldwide on various hosts within terrestrial habitats (see Table 2). Specifically, it has been documented in Australia (*Calamusaustralis*, *Vitisvinifera*), Brazil (*Bambusoideae*, *Saccharumofficinarum*), Central African Republic (*Coffearobusta*), China (*Althaearosea*, *Craterellusodoratus*, *Trachycarpusfortunei*), Colombia (unknown plant), France (*Pseudosasajaponica*), Georgia (*Zeamays*), India (*Acacia* sp., *Adhatodavasica*, *Ailanthusaltissima*, *Annonasquamosa*, *Cajanuscajan*, *Careyaarborea*, *Citrusaurantiifolia*, *Clerodendruminfortunatum*, *C.multiflorum*, *Durantarepens*, *Ficusglomerata*, *Hibiscus* sp., *Ipomoeacarnea*, *Mallotusphilippinensis*, *Morusalba*, *Neriumodorum*, *Pistaciaindica*, *Tectonagrandis*, *Terminaliatomentosa*), Japan (*Phyllostachysbambusoides*), Myanmar (*Nepheliumlitchi*), Namibia (*Acaciareficiens*), Papua New Guinea (*Bambusoideae*), Paraguay (*Coffeaarabica*), Philippines (*Premnacumingiana*), Portugal (*Arundodonax*), Sierra Leone (*Funtumiaafricana*), Thailand (dead wood) and the USA (*Platanus* sp., *Wikstroemia* sp.).

##### Material examined.

China, Yunnan Province, Honghe (23.424892°N, 102.231417°E, 600 m), on dead woody litter of an unidentified plant, 14 August 2022, D.N. Wanasinghe, DWHH22-23-1 (HKAS 126545), living culture KUNCC 23-14428. *ibid*. DWHH22-23-2 (HKAS 126546), living culture KUNCC 23-14429.

##### Notes.

Wanasinghe et al. (2016) regarded *Munkovalsaria* as a synonym of *Montagnula* and established *Montagnuladonacina* (=*Munkovalsariadonacina*). So far, *Montagnuladonacina* stands as the most extensively distributed species within the genus. Despite its global presence, there is a scarcity of molecular data available for *Montagnuladonacina*. A preliminary analysis revealed only 20 sequence data entries when searching for “ *Montagnuladonacina*” in the NCBI database, originating from only seven strains: HFG07004, HKAS 124552, HVVV01, KUMCC 21-0579, KUMCC 21-0631, KUMCC 21-0653, and UESTCC:23.0030. Our phylogenetic analysis demonstrated a close relationship between two strains designated as *Montagnuladonacina* (HVVV01 and HFG07004) and the type strain of *Montagnulachromolaenicola* (MFLUCC 17-1469). Additionally, we observed that the strains of *Montagnulathailandica* formed a monophyletic group alongside the remaining *Montagnuladonacina* strains (HKAS 124552, KUMCC 21-0579, KUMCC 21-0631, KUMCC 21-0653, and UESTCC:23.0030). Furthermore, two newly generated sequences, KUNCC 23-14428 and KUNCC 23-14429, were also clustered with the strains of *Montagnuladonacina*. We hereby introduce these two strains as belonging to *Montagnuladonacina* and provide *rpb*2 sequence data for this species for the first time.

#### ﻿ Montagnula lijiangensis

Taxon classificationFungiPleosporalesDidymosphaeriaceae

Wanas. sp. nov.

9744156B-D833-5D0D-93D2-347AE9C73247

850093

Fig. 6

##### Etymology.

The specific epithet “lijiangensis” refers to Lijiang, Yunnan Province, where the holotype was collected.

##### Holotype.

HKAS 126541.

##### Description.

***Saprobic*** on dead woody litter of *Quercus* sp. **Teleomorph *Ascomata*** 500–700 μm high × 500–600 μm diam., immersed, gregarious or rarely clustered, globose to subglobose, ostiolate. ***Ostiole*** 100–140 × 80–120 µm (x– = 125 × 96 μm, n = 5), apapillate, central, straight, filled with hyaline cells. ***Peridium*** 20–30 μm thin on the sides and can reach up to 70 μm near the apex, with an outer layer consisting of heavily pigmented cells that have thick walls and exhibit a ***textura angularis*** texture at the apex, ***textura angularis*** texture at the sides and base; the innermost layer consists of narrow, hyaline compressed rows of cells. ***Hamathecium*** of 3–7.5 μm broad, dense, narrow, branched, cellular pseudoparaphyses that are swollen at the base. ***Asci*** 130–160 × 20–26 µm (x– = 152.8 × 23.9 μm, n = 20), bitunicate, fissitunicate, cylindrical-clavate to clavate, pedicel 30–60 μm long, 8-spored, uni to biseriate, with a minute ocular chamber best seen in immature ascus. ***Ascospores*** 22–26 × 10–14 µm (x– = 24.8 × 11.8 μm, n = 30), ellipsoidal to narrowly oblong, mostly straight, with conically rounded ends at the immature stage that become rounded when mature, golden-brown to dark brown, 1-septate and constricted at the septum, with large guttules in each cell, verruculose, surrounded by a thick mucilaginous sheath. **Anamorph** Undetermined.

**Figure 6.**
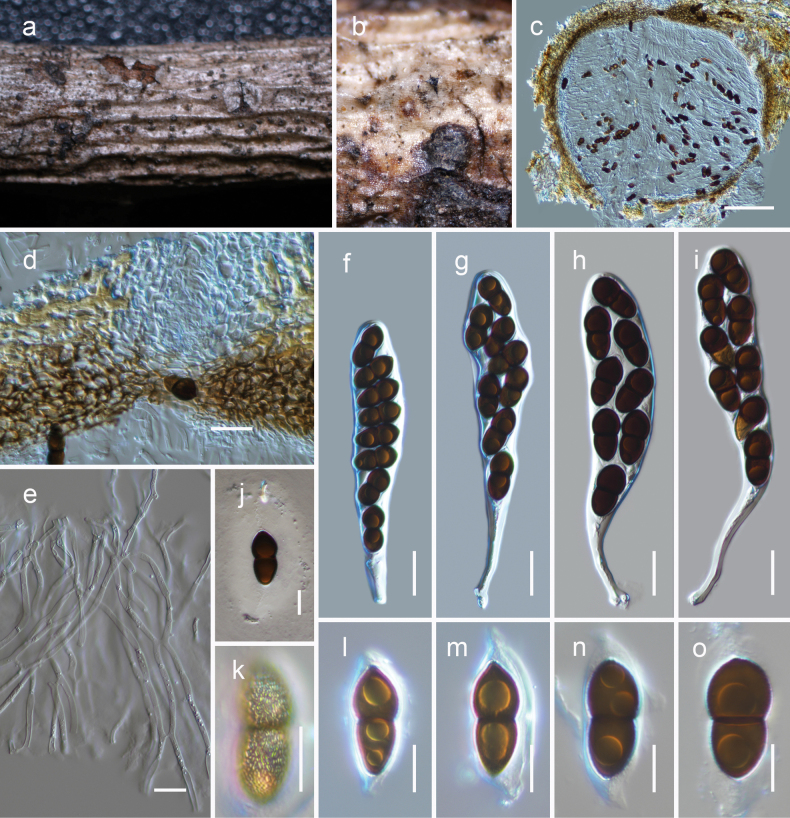
*Montagnulalijiangensis* (HKAS 126541, holotype) **a, b** ascomata on natural wood surface **c** vertical section through an ascoma **d** ostiolar neck and peridium cells at the apex **e** pseudoparaphyses **f–i** asci **j–o** ascospores (see verruculose feature of the ascospore in **k**). Scale bars: 100 μm (**c**); 20 μm (**d, f–i**); 10 μm (**e–o**).

##### Habitat and distribution.

This species is found in terrestrial habitats of Yunnan, China, inhabiting dead woody twigs of deciduous hosts (this study).

##### Material examined.

China, Yunnan Province, Lijiang, Yulong County (26.86389°N, 99.824738°E, 2725 m), on dead woody litter of *Quercus* sp. (Fagaceae), 17 August 2021, L. Qinxian, STX09-03-1 (***holotype***, HKAS 126541, *ibid*. 26.863484°N, 99.824548°E, 2706 m, STX09-03-3 (HKAS 126540).

##### Notes.

The analysis of two newly generated sequences revealed a monophyletic clade in our phylogenetic analysis (Fig. 1), demonstrating a close phylogenetic relationship to *Montagnulaaquilariae*. This relationship is further supported by morphological features such as asci and ascospores. However, a comparison of nucleotide differences (without gaps) between these two clades (KUNCC 22-10815 and KUNCC 23-14430 vs HKAS 126541) showed 12/508 (2.3%) differences in the ITS region, 15/885 (1.7%) differences in the *tef*1-α region, and 19/956 (2%) differences in the *rpb*2 region.

#### ﻿ Montagnula menglaensis

Taxon classificationFungiPleosporalesDidymosphaeriaceae

Wanas. sp. nov.

E04FC54E-5CD2-59B6-8BFC-75EEE1F17429

850094

Fig. 7

##### Etymology.

The specific epithet “menglaensis” refers to Mengla County, Yunnan Province, where the holotype was collected.

##### Holotype.

HKAS 130318.

##### Description.

***Saprobic*** on dead culms of *Indocalamustessellatus* (Munro) Keng f. **Teleomorph *Ascomata*** 200–300 μm high × 240–320 μm diam., immersed, gregarious or rarely clustered, globose to subglobose. ***Peridium*** 10–25 μm thin with an outer layer consisting of heavily pigmented cells that have thick walls and exhibit a ***textura angularis*** texture at the sides and base; the innermost layer consists of narrow, hyaline compressed rows of cells. ***Hamathecium*** of 3–7.5 μm broad, dense, branched, cellular pseudoparaphyses that are swollen at some septa. ***Asci*** 60–80 × 9–11 µm (x– = 71 × 9.8 μm, n = 15), bitunicate, fissitunicate, cylindrical-clavate, pedicel 15–30 μm long, 8-spored, uni to biseriate, with a minute ocular chamber best seen in immature ascus. ***Ascospores*** 10.5–14 × 4.5–5.5 µm (x– = 12.6 × 5.1 μm, n = 20), ellipsoidal, mostly straight, with conically rounded ends, golden-brown to dark brown, 1-septate and constricted at the septum, upper cell wider than the lower cell, with large guttules in each cell, verruculose, and surrounded by a thin mucilaginous sheath which is thicker at both ends. **Anamorph** Coelomycetous on PDA. ***Conidiomata*** pycnidial, gregarious, immersed to superficial, globose to subglobose, dark brown to black. ***Pycnidial wall*** thin, composed of brown cells of ***textura angularis***. ***Conidiogenous cells*** did not observed. ***Conidia*** 2.3–3.3 × 1.4–2 μm (x– = 3 × 1.7 μm, n = 30), hyaline, aseptate, round to oblong or ellipsoidal, with small guttules.

**Figure 7.**
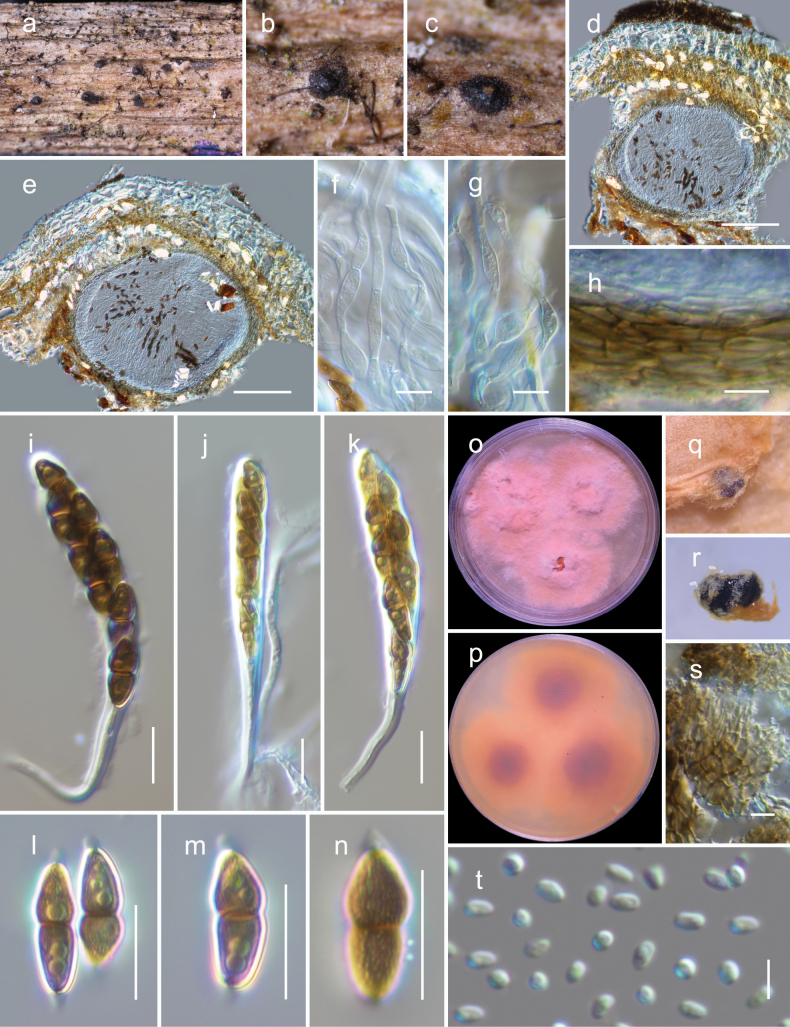
*Montagnulamenglaensis* (HKAS 130318, holotype) **a–c** ascomata on natural wood surface **d, e** vertical section through ascomata **f, g** pseudoparaphyses **h** peridium **i–k** asci **l, m** ascospores (see verruculose feature of the ascospore in **n**) **o, p** culture characters on PDA (o = above, p = reverse) **q, r** conidiomata **s** pycnidial wall **t** conidia. Scale bars: 100 μm (**d, e**); 10 μm (**f–h, l–n, s, t**); 20 μm (**i–k**).

##### Culture characteristics.

Ascospores germinated on PDA within 24 h. Following a two-week incubation period at 25 °C, the colonies on PDA medium reached a diameter of 5 cm. These colonies exhibited an undulate margin, initially appearing creamy whitish and transitioning to orange, raised in the center. The colonies were orange at the center and a creamy orange towards the periphery when observed from the reverse side.

##### Habitat and distribution.

This species is found in terrestrial habitats of Yunnan, China, inhabiting dead woody twigs of deciduous hosts (this study).

##### Material examined.

China, Yunnan Province, Xishuangbanna, Mengla County (21.588394°N, 101.435042°E, 776 m), on dead culms of *Indocalamustessellatus*, 29 January 2022, L. Qinxian, ML23-7-3 (holotype, HKAS 130318), ex-type KUNCC 23-14424; *ibid*. 21.589178°N, 101.435752°E, 782 m, ML23-7-2 (HKAS 130316), living culture KUNCC 23-14422; *ibid*. ML23-7-5 HKAS 130317), living culture KUNCC 23-14423.

##### Notes.

*Montagnulamenglaensis* is described as a novel species based on its holomorph. The anamorph of *Montagnula* is rarely encountered; however, Crous et al. (2020) recently reported *Montagnulacylindrospora* based on its anamorphic features. The conidia of *Montagnulamenglaensis* resemble to those of *M.cylindrospora*, although the latter fungus exhibits a more cylindrical shape.

#### ﻿ Montagnula shangrilana

Taxon classificationFungiPleosporalesDidymosphaeriaceae

Wanas. sp. nov.

BD5D6130-3E22-55BD-BBF2-DB95AD1C1DA2

850095

Fig. 8

##### Etymology.

The specific epithet “shangrilana” refers to Shangri-La, Yunnan Province, where the holotype was collected.

##### Holotype.

HKAS 126539.

##### Description.

***Saprobic*** on dead woody litter of *Rhododendron* sp. **Teleomorph *Ascomata*** 120–180 μm high × 150–210 μm diam., immersed to semi-erumpent, gregarious or rarely clustered, globose to subglobose, ostiolate. ***Ostiole*** 80–110 × 50–80 µm (x– = 100 × 64 μm, n = 6), papillate, central, straight, filled with hyaline cells. ***Peridium*** 10–20 μm thin on the sides and can reach up to 40 μm near the apex, with an outer layer consisting of heavily pigmented cells that have thick walls and exhibit a ***textura angularis*** arrangement at the apex, ***textura angularis*** texture at the sides; the innermost layer consists of hyaline compressed rows of cells. ***Hamathecium*** of 2–4.5 μm broad, dense, branched, cellular pseudoparaphyses. ***Asci*** 90–140 × 20–30 µm (x– = 116.2 × 24 μm, n = 10), bitunicate, fissitunicate, cylindrical-clavate, pedicel 25–40 μm long, 8-spored, uni to biseriate, with a minute ocular chamber best seen in immature ascus. ***Ascospores*** 48–60 × 17–22 µm (x– = 55.8 × 19.3 μm, n = 20), ellipsoidal to narrowly oblong, mostly straight, with conically rounded ends at the immature stage that become rounded when mature, golden-brown to dark brown, 3-septate, with large guttules in each cell, verruculose, surrounded by a thick mucilaginous sheath. **Anamorph** Undetermined.

**Figure 8.**
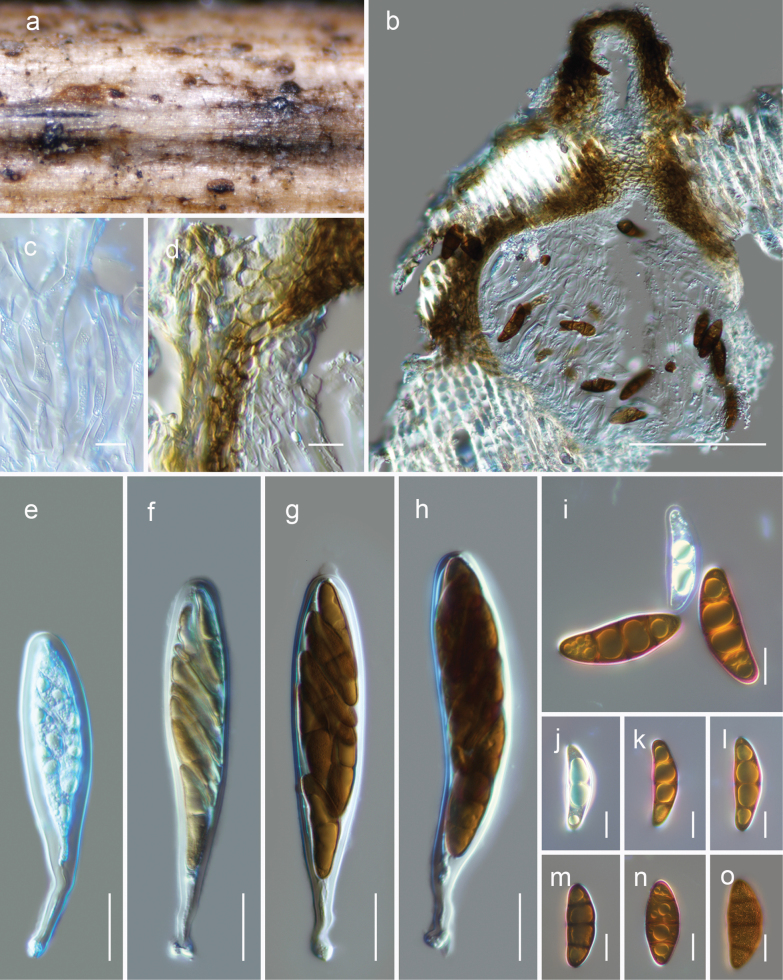
*Montagnulashangrilana* (HKAS 126541, holotype) **a** ascomata on natural wood surface **b** vertical section through an ascoma **c** pseudoparaphyses **d** peridium cells **e–h** asci **i–o** ascospores (see verruculose feature of the ascospore in **o**). Scale bars: 100 μm (**b**); 10 μm (**c, d, j–o**); 20 μm (**e–h**).

##### Culture characteristics.

Ascospores germinated on PDA within 24 h. Following a two-week incubation period at 25 °C, the colonies on PDA medium reached a diameter of 5 cm. These colonies exhibited a filiform margin, initially appearing whitish and transitioning to greenish gray, raised in the center. The colonies were grey at the center and a greenish gray towards the periphery and radiated when observed from the reverse side.

##### Habitat and distribution.

This species is found in terrestrial habitats of Yunnan, China, inhabiting dead woody twigs of deciduous hosts, in a subalpine environment (this study).

##### Material examined.

China, Yunnan Province, Diqing Tibetan Autonomous Prefecture, Shangri-La (27.289707°N, 100.034477°E, 2744 m), on dead woody litter of *Rhododendron* sp. (Ericaceae), 22 August 2021, L. Qinxian, WTS8-2-2 (holotype, HKAS 126539), ex-type KUNCC 23-14434; *ibid*. (27.290007°N, 100.035233°E, 2833 m, WTS8-2 (HKAS 126538), living culture KUNCC 23-14433.

##### Notes.

In the combined SSU, LSU, ITS, *tef*1-α, and *rpb*2 phylogenetic analysis, two strains of *Montagnulashangrilana* (HKAS 126538, HKAS 126539) formed a monophyletic clade closely related to *M.camporesii* (MFLUCC 16-1369), *M.cirsii* (MFLUCC 13-0680), and *M.scabiosae* (MFLUCC 14-0954). While there were slight variations in size, shape, and color, all four species shared the common characteristic of 3-transversely septate ascospores. The sequence data of *Montagnulacamporesii*, *M.cirsii*, and *M.scabiosae* showed no significant differences in their base pair comparisons, suggesting that they may be conspecific. Morphologically, these three species exhibited clavate asci and ellipsoid to fusiform, brown, overlapping, 3-septate ascospores. In contrast, our newly discovered species differed from these three species by 10/508 (1.96%) differences in the ITS region, 13/885 (1.5%) differences in the *tef*1-α region, and 15/956 (1.56%) differences in the *rpb*2 region (only *M.camporesii* possesses *rpb*2).

#### ﻿ Montagnula thevetiae

Taxon classificationFungiPleosporalesDidymosphaeriaceae

Wanas. sp. nov.

03DE911B-99C1-5A3C-AB33-13DA09C0F3AA

850096

Fig. 9

##### Etymology.

The specific epithet “thevetiae” refers to the host *Thevetiaperuviana* from which the holotype was isolated.

##### Holotype.

HKAS 126964.

##### Description.

***Saprobic*** on dead twigs of *Thevetiaperuviana*. **Teleomorph *Ascomata*** 140–160 μm high × 150–190 μm diam., immersed, gregarious or rarely clustered, globose to subglobose, ostiolate. ***Ostiole*** 40–65 × 50–90 µm (x– = 50 × 78 μm, n = 6), papillate, central, straight, filled with hyaline to brown cells. ***Peridium*** 10–20 μm thin on the sides and can reach up to 30 μm near the apex, with an outer layer consisting of heavily pigmented cells that have thick walls and ***textura angularis*** arrangement, the inner layer consists of hyaline compressed rows of cells. ***Hamathecium*** of 2–3.5 μm broad, dense, branched, cellular pseudoparaphyses. ***Asci*** 110–160 × 25–35 µm (x– = 126.4 × 30.3 μm, n = 12), bitunicate, fissitunicate, cylindrical-clavate, pedicel 25–35 μm long, 8-spored, uni to biseriate, with a minute ocular chamber best seen in immature ascus. ***Ascospores*** 30–40 × 11.5–14 µm (x– = 37.3 × 12.8 μm, n = 20), ellipsoidal to narrowly oblong, straight to curved, with conically rounded ends, brown to dark brown, 1-septate, constricted at the septum, with large guttules in each cell, verruculose, surrounded by a thin mucilaginous sheath. **Anamorph** Undetermined.

**Figure 9.**
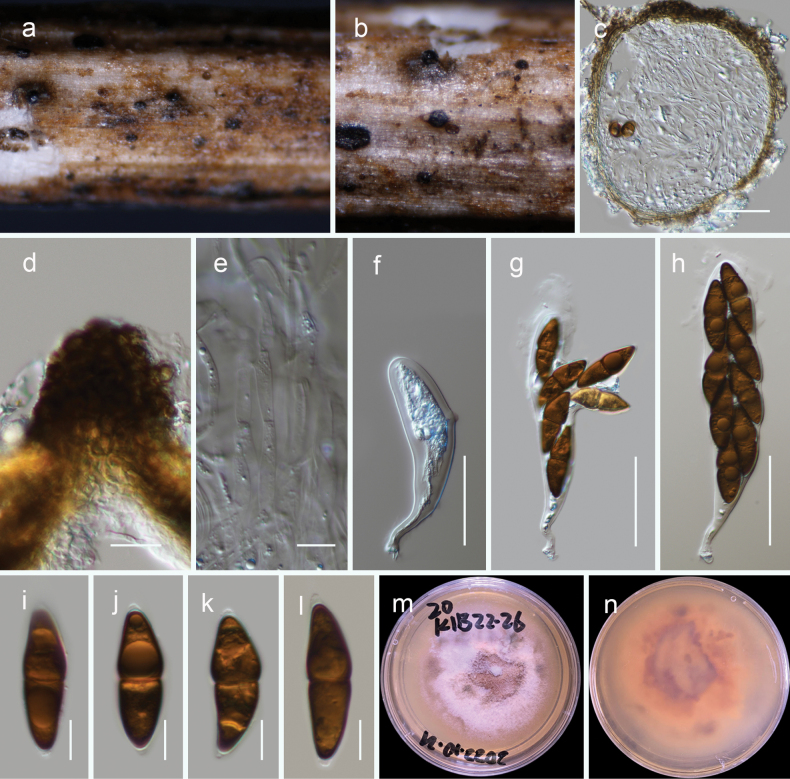
*Montagnulathevetiae* (HKAS 126564, holotype). **a, b** ascomata on natural wood surface **c** vertical section through an ascoma **d** closeup of ostiole **e** pseudoparaphyses **f–h** asci **j–l** ascospores **m, n** culture characteristics on PDA (m = above, n = reverse). Scale bars: 100 μm (**c**); 50 μm (**d, f–h**); 10 μm (**e, i–l**).

##### Culture characteristics.

Ascospores germinated on PDA within 24 h. Following a two-week incubation period at 25 °C, the colonies on PDA medium reached a diameter of 4 cm. These colonies exhibit an irregular, flattened to slightly raised morphology and display various color sectors ranging from white, creamy orange to pale brown. The reverse side of the colonies appears creamy orange, with occasional dark patches that can be observed.

##### Habitat and distribution.

This species is found in terrestrial habitats of Yunnan, China, inhabiting dead woody twigs of *Thevetiaperuviana* (this study).

##### Material examined.

China, Yunnan Province, Kunming city, Kunming Institute of Botany (25.142238°N, 102.750354°E, 1971 m), on dead twigs of *Thevetiaperuviana*, 24 April 2022, L. Qinxian, K2B22-26-2 (holotype, HKAS 126964), *ibid*. (25.140859°N, 102.749045°E, 1968 m, K2B22-26 (HKAS 126963).

##### Notes.

*Montagnulathevetiae* is isolated from the dead twigs of *Thevetiaperuviana*. The newly obtained sequences of this fungus formed a monophyletic clade closely related to *Montagnulamenglaensis*. Morphologically, they share similarities in having 1-septate ascospores, although *Montagnulathevetiae* exhibits a darker pigmentation. On the other hand, *Montagnulathevetiae* differs from *M.menglaensis* by 15/1023 (1.46%) differences in the SSU region, 19/895 (2.12%) differences in the LSU region, 32/508 (6.3%) differences in the ITS region, 27/885 (3%) differences in the *tef*1-α region, and 86/956 (9%) differences in the *rpb*2 region.

## ﻿Discussion

### ﻿*Montagnula* species in Yunnan Province

The study of lignicolous microfungi in Yunnan Province resulted in the collection of eight *Montagnula* species, including four novel species. This study contributes to our understanding of the diversity and distribution of *Montagnula* species and provides insight into the ecological roles played by these fungi in their respective habitats. *Montagnulaaquatica* was previously documented as occurring on submerged decaying wood within a freshwater habitat in Thailand (Sun et al. 2023). However, our recent collection of this species was obtained from a terrestrial habitat in China. The holotype was collected in the Bandu District of the Chiang Rai Province, situated at an approximate elevation of 400–450 m and characterized by a tropical climate. The collection site was near to a waterfall (Sun et al. 2023). In contrast, our new collections were made in the Honghe region of Yunnan Province, which possesses an elevation of approximately 1200 m. The local environment in this region is characterized by poor, eroded soils, steep valleys, and a subtropical climate. This observation suggests that *Montagnulaaquatica* may possess an adaptable nature, enabling it to thrive in a wide range of habitats across diverse geographic locations. *Montagnulaaquilariae*, another species within the genus, has been identified in the terrestrial habitats of Yunnan, China. It specifically colonizes dead woody twigs of deciduous hosts, including *Aquilariasinensis* (Hyde et al. 2023). The holotype of this species was collected from a hilly area in Nanmo, Menghai and Xishuangbanna, situated at an elevation of ~1100 m and characterized by a tropical climate. Additional collections were made from Kunming, located within the same province but at an elevation of ~2000 m, and characterized by a warm and temperate climate. *Montagnulachromolaenicola* has been observed in terrestrial habitats in Thailand, particularly on dead stems of *Chromolaenaodorata* (Mapook et al. 2020). The holotype of this species was collected from the Mae Yen mountainous area of Mae Hong Son Province, at an elevation of ~900 m. The local environment of this area exhibits a tropical savanna climate. In our study, we collected this fungus from a terrestrial habitat within the steep valleys of subtropical Honghe, Yunnan, China. In this region, *Montagnulachromolaenicola* was found to inhabit the dead woody litter of deciduous hosts. *Montagnuladonacina* has been reported across various terrestrial habitats worldwide, with the majority of records originating from India (Table 1). This species primarily associates with hosts from the Poaceae family. In our study, we collected *Montagnuladonacina* from the subtropical Honghe region in China, specifically on decaying woody litter at an elevation of ~600 m. *Montagnulalijiangensis* was collected from terrestrial habitats at a high elevation of ~2725 m. This species was found on dead woody litter of *Quercus* sp. within an environment characterized by a mild subtropical highland climate. *Montagnulamenglaensis* was discovered in the terrestrial habitats of Mengla County, Yunnan, China. It was observed colonizing dead culms of *Indocalamustessellatus*. The local environment of this region exhibits a tropical savanna climate, with an elevation of ~800 m. *Montagnulashangrilana* was found in the terrestrial habitats of Shangri-La, Yunnan, China, where it inhabits dead woody twigs of *Rhododendron* sp. This species has also been observed at higher elevations, reaching ~2800 m, within an environment characterized by a humid continental climate. *Montagnulathevetiae* was discovered within the terrestrial habitat of the botanical garden at the Kunming Institute of Botany in Yunnan, China. This species was found colonizing dead woody twigs of *Thevetiaperuviana*. The collection site is situated at an elevation of ~2000 m and experiences a warm and temperate climate.

### ﻿Taxonomic reassessment and phylogenetic analysis of *Montagnula* species

In a recent study conducted by Sun et al. (2023), *Montagnulachromolaenicola*, *M.puerensis*, *M.saikhuensis*, and *M.thailandica* were regarded as the synonyms of *M.donacina* (Wanasinghe et al. 2016). This decision was based on the absence of clear branches in their phylogenetic tree and the close morphological resemblance between these species. However, upon further examination, it was observed in this study that only *Montagnuladonacina* and *M.thailandica* appear to be conspecific, based on combined gene analyses (Fig. 1). When informative sequence data such as *tef*1-α or *rpb*2 were added to the analysis for *Montagnulachromolaenicola*, *M.puerensis*, *M.saikhuensis*, and *M.thailandica*, distinct branches and independent lineages were observed (Fig. 1). This suggests that these species are separate entities. Notably, two sequences of *M.donacina* (HVVV01 and HFG07004) were found to be monophyletic with the type strain of *Montagnulachromolaenicola* (MFLUCC 17-1469), indicating that they belong to the latter species. In the case of *Montagnulacamporesii* (MFLUCC 16-1369), *M.cirsii* (MFLUCC 13-0680), and *M.scabiosae* (MFLUCC 14-0954), the type strains formed a monophyletic lineage as a single species. Nucleotide base pair comparison of LSU, SSU, and ITS between these three strains did not reveal any differences. Therefore, it is suggested that *Montagnulacamporesii* and *M.cirsii* should be synonymized under *M.scabiosae*, as it is the oldest name. However, it is important to note that this taxonomic clarification was not within the scope of our study, and future studies should compare the morphology of the holomorphs to resolve any remaining taxonomic confusion. Apart from these two clades, all other species formed distinct lineages in the multi-gene phylogenetic analysis. Out of the accepted 54 species in this genus, sequence data are currently available for only 28 species, including the four new species introduced in this study. This leaves approximately 48% of the species in need of phylogenetic sorting. Hence, future studies based on taxonomy should prioritize obtaining DNA sequence data for the remaining species. They should aim to acquire informative sequence data, such as *tef*1-α and *rpb*2, for all strains, and focus on revising the taxonomy of all species within the genus *Montagnula*.

### ﻿Morphological characterization of *Montagnula* species

The genus *Montagnula* exhibits rare reporting of its anamorphic features, with only one species, *M.cylindrospora*, described from its anamorph in addition to our study (Crous et al. 2020). This finding has helped confirm its phylogenetic placement within the genus. The teleomorph, rather than the anamorph, appears to be more commonly observed in the natural environment. The majority of *Montagnula* species produce immersed or semi-immersed ascomata, which are globose to subglobose in shape and possess a central papillate ostiole. However, there are a few exceptions, such as *M.camporesii*, *M.cirsii*, and *M.longipes*, which have been reported to have superficial ascomata. Upon closer examination, it becomes apparent that *Montagnulacamporesii* and *M.cirsii* actually have semi-immersed ascomata, as illustrated in Hyde et al. (2016, 2020). It is worth mentioning that Aptroot (1995) did not illustrate the ascomata, and their orientation remains unclear. Additionally, only one species, *Montagnulabellevaliae*, has been reported to possess an eccentric ostiole (Hongsanan et al. 2015). The peridium cells of *Montagnula* species commonly exhibit a thick-walled arrangement with a textura angularis pattern. Notably, the cells near the apex are often thicker compared to those on the sides and base walls. A distinguishing characteristic for species within this genus is the presence of swollen cells in pseudoparaphyses. The asci, typically exhibit a cylindrical to clavate shape with a prominent pedicel. Ascospores in *Montagnula* are predominantly described as ellipsoidal to fusiform, pigmented, and septate. The majority of species (>15) have ascospores with a single septum, while some species, including *M.dasylirionis*, *M.dura*, *M.infernalis*, *M.mohavensis*, *M.phragmospora*, *M.spinosella*, and *M.yuccigena*, have been reported to possess muriform spores (Du et al. 2023). The remaining species have ascospores with either 3 or 5 septa. A distinct characteristic within the genus is the verruculose surface texture of the ascospores which is neglected by most of the studies. Only *Montagnulaappendiculata*, *M.chiangraiensis*, and *M.chromolaenae* have been documented to possess polar appendages (Aptroot 2004; Mapook et al. 2020).

### ﻿Ecological preferences and worldwide distribution of *Montagnula* species through culture-dependent studies

The information we gathered from our culture-based investigations revealed that *Montagnula* species were found on 105 genera in 45 distinct plant families, in 55 countries (Table 1). This highlights the wide ecological range and adaptability of *Montagnula* species across different hosts and geographic regions. Among the plant families, Poaceae emerged as the most significant contributor, yielding the highest number of isolated *Montagnula* species (Fig. 4). This finding suggests a potential association between *Montagnula* species and grasses, indicating the ecological importance of the Poaceae family in the life cycle and development of *Montagnula* species. Furthermore, *Montagnula* species were also detected in other plant families, such as Asparagaceae and Fabaceae, indicating their potential interactions with a diverse range of host plants. Among the more than 100 plant genera associated with *Montagnula* species, *Agave* (Asparagaceae), *Opuntia* (Cactaceae), *Phoenix* (Arecaceae), *Ammophila* (Poaceae), and *Yucca* (Asparagaceae) were found to have the greatest number of species, collectively representing 25% of the total count. This highlights the potential preference of *Montagnula* species for these specific plant genera within their respective families. The analysis of country-wise distribution revealed that India had the highest number of *Montagnula* entries (Table 1). The majority of these entries were attributed to *Montagnuladonacina*, indicating a wide distribution of this species in India. Among the countries where *Montagnula* species were reported, China exhibited the highest diversity with nine different species, followed by Italy and the USA with seven different species each. This suggests regional variations in the diversity and distribution of *Montagnula* species. Interestingly, our study also detected *Montagnula* species in mushrooms and human skin samples, indicating their presence in alternative sources and potential interactions with other organisms. This highlights the need for further investigation into the ecological roles and potential impacts of *Montagnula* species in these non-traditional habitats. Except for Antarctica, *Montagnuladonacina* has been reported from various countries across all six continents. Additionally, it has been identified in 25 different plant families. Investigating the reasons behind its wide distribution and adaptation to diverse ecological conditions would be intriguing. Future studies should focus on the morphological features, secondary metabolites, and gene data-based analyses of the species. To date, only six studies, including this one, have provided entries featuring both morphology and DNA-based sequence data evidence (Pitt et al. 2014; Zhao et al. 2018; Ren et al. 2022a; Li et al. 2023; Sun et al. 2023).

These findings elucidate the global distribution and ecological preferences of *Montagnula* species, highlighting the significance of different sources and plant families in their occurrence and potential ecological interactions. The wide range of sources from which species were identified suggests their adaptability and potential ecological roles in various ecosystems. The study also has important implications for our understanding of the ecology and biology of *Montagnula* fungi. All of the new species described in this study were found to be associated with dead wood, indicating the role that these fungi play in the decomposition of organic matter in forest ecosystems. We suggest that future studies could investigate the functional roles played by *Montagnula* fungi in ecosystem processes, such as carbon and nutrient cycling.

### ﻿Global biogeography and ecological versatility of *Montagnula* based on metabarcoding data through culture-independent studies (NGS)

In addition to the taxonomic novelties, this study utilized metabarcoding data from the GlobalFungi database (Větrovský et al. 2020) to gain insights into the global diversity and distribution of *Montagnula*. Metabarcoding is a valuable tool that allows for the rapid identification of multiple species from complex environmental samples, providing confirmation of their presence in specific habitats. The analysis of multiple metabarcoding studies provided comprehensive information on the occurrence and distribution patterns of *Montagnula* species worldwide. The distribution of *Montagnula* across diverse biomes underscores their remarkable ecological adaptability and diversity. Forests, constituting 61% of their habitats, emerge as the predominant biome, indicating a strong preference or adaptation of the genus to forest ecosystems. Grasslands, accounting for 18%, also represent a significant habitat, suggesting the versatility in adapting to open and semi-open landscapes of them. Croplands (6%) and shrublands (7%) further exemplify the adaptability of *Montagnula*, thriving in both cultivated areas and natural, low-vegetation environments. Notably, woodlands and anthropogenic areas, representing 2% and 1% respectively, highlight the ability to exist in moderately wooded areas and regions significantly influenced by human activity. Additionally, their presence in aquatic environments, deserts, and wetlands, each accounting for 1% of their habitats, along with a notable 3% in mangroves, reflects the broad ecological niche of them. The marginal occurrence in tundras (0.1%) suggests a limited but notable ability to survive in extreme cold climates. The presence of *Montagnula* in such varied biomes underscores its ecological versatility and the importance of diverse habitats in understanding its biogeography.

The presence of *Montagnula* species has been documented in various regions of Africa, Arctic Ocean, Asia, Australia, Europe, Indian Ocean, North America, Pacific Ocean and South America indicating their widespread occurrence and ecological significance in these areas. In Asia, *Montagnula* species have been observed in multiple countries, including China, India, Indonesia, Iran, Japan, Malaysia, South Korea, Thailand and others (Suppl. material 1). The diverse range of habitats in these regions, such as freshwater habitats, terrestrial environments, and mountainous areas, offer suitable ecological niches for *Montagnula* colonization and growth. The detection of *Montagnula* species in different ecological contexts within Asia suggests their ability to adapt to various local conditions and substrates, contributing to their wide distribution across the continent. For example, in China, *Montagnula* species have been found in diverse habitats ranging from aquatic environments to forests and grasslands (Suppl. material 1), indicating their adaptability to different ecosystems. This adaptability may be attributed to their ability to utilize a wide range of organic materials as substrates, including decaying plant remains.

Australia also exhibits a notable presence of *Montagnula* species, indicating their occurrence in diverse habitats throughout the continent (Bissett et al. 2016; Luis et al. 2019; Turner et al. 2019; Gui et al. 2023). The unique ecosystems in Australia, including deserts, rainforests and grasslands, provide opportunities for *Montagnula* to establish themselves in different ecological niches. The metabarcoding studies were used for various biomes i.e. anthropogenic, aquatic, cropland, desert, forest, grassland, mangrove, shrubland, wetland and woodland (Fig. 3). This highlights the higher presence and distribution of *Montagnula* in different habitats within Australia. In Europe, *Montagnula* species have been recorded in several countries, including Austria, Belgium, Czech Republic (highest), Estonia, France, Germany, Italy, Netherlands Slovenia, Sweden Switzerland and Spain (Suppl. material 1). The presence of *Montagnula* in Europe suggests their ability to adapt to different climates and ecological conditions. This broad distribution across Europe indicates the need for further investigation into the ecological preferences and potential impacts of *Montagnula* species in this region. For instance, studies in Europe have identified *Montagnula* species in different habitats, such as anthropogenic, aquatic, cropland, desert, forest, grassland, shrubland, tundra, wetland and woodland (Suppl. material 1). Africa and North America also demonstrates a diverse distribution of *Montagnula* species, with the majority of records coming from the South Africa, Namibia, Botswana, Zambia, Mozambique, Kenya, Kenya and Ivory Coast in Africa respectively. United States was having the highest number of sampling locations in North America. Comparatively, the occurrences of *Montagnula* species using metabarcoding data in China, the USA, and European countries are relatively well-documented. However, the rest of the world remains a mystery in terms of *Montagnula* distribution. For example, the majority of Asia, including India and Russia, lacks metabarcoding data for *Montagnula* species. This emphasizes the need for more extensive research and data collection to better understand the global distribution of *Montagnula* and its ecological roles.

## ﻿Conclusion

Our study on *Montagnula* species has provided valuable insights into their ecological preferences and global distribution patterns. The findings indicate that these fungi exhibit a wide range of climatic distribution, suggesting their adaptability to different temperature ranges and potentially reducing their vulnerability to climate change. The ability of *Montagnula* species to utilize a diverse range of organic materials as substrates, including decaying plant remains, contributes to their widespread distribution across various habitats. Our analysis revealed a diverse range of sources from which *Montagnula* species were detected, including freshwater and terrestrial habitats, further highlighting their ecological versatility. Sediments were found to be particularly rich in *Montagnula* sequences, suggesting their potential as suitable habitats for colonization and growth. Although moderate sequence similarity was observed across different sources and continents, regional variations in ecological preferences and distribution patterns were evident. The diverse host range observed in our field collections aligns with global meta-barcoding sources, emphasizing the ability of *Montagnula* species to thrive in various ecosystems. The ecological adaptability and versatility of *Montagnula* species underscore their success in colonizing diverse habitats. Further research and investigation into their biogeography will contribute to our understanding of their global distribution, ecological roles, and potential impacts on ecosystems. This knowledge is crucial for effective conservation efforts, understanding ecosystem dynamics, and managing ecological balance in different regions.

## Supplementary Material

XML Treatment for Montagnula

XML Treatment for Montagnula aquatica

XML Treatment for Montagnula aquilariae

XML Treatment for Montagnula chromolaenicola

XML Treatment for Montagnula donacina

XML Treatment for Montagnula lijiangensis

XML Treatment for Montagnula menglaensis

XML Treatment for Montagnula shangrilana

XML Treatment for Montagnula thevetiae

## ﻿Additional information

### Author ORCIDs

Dhanushka N. Wanasinghe https://orcid.org/0000-0003-1759-3933

Thilina S. Nimalrathna https://orcid.org/0000-0002-2368-042X

Li Qin Xian https://orcid.org/0009-0006-4936-9409

Turki KH. Faraj https://orcid.org/0000-0002-6012-8474

Jianchu Xu https://orcid.org/0000-0002-2485-2254

Peter E. Mortimer https://orcid.org/0000-0003-3188-9327

### Data availability

All of the data that support the findings of this study are available in the main text or Supplementary Information.

## Supplementary materials

Supplementary material 1 The biogeography, substrate and habitat affinity of *Montagnula* inferred from the GlobalFungi database Data type xlsx https://binary.pensoft.net/file/970524This dataset is made available under the Open Database License (http://opendatacommons.org/licenses/odbl/1.0/). The Open Database License (ODbL) is a license agreement intended to allow users to freely share, modify, and use this Dataset while maintaining this same freedom for others, provided that the original source and author(s) are credited.Dhanushka N. Wanasinghe, Thilina S. Nimalrathna, Li Qin Xian, Turki KH. Faraj, Jianchu Xu, Peter E. Mortimer
